# Systematic Engineering of *Saccharomyces cerevisiae* for the De Novo Biosynthesis of Genistein and Glycosylation Derivatives

**DOI:** 10.3390/jof10030176

**Published:** 2024-02-26

**Authors:** Yongtong Wang, Zhiqiang Xiao, Siqi Zhang, Xinjia Tan, Yifei Zhao, Juan Liu, Ning Jiang, Yang Shan

**Affiliations:** 1 Longping Branch, College of Biology, Hunan University, Changsha 410125, China; quarknorthstar@163.com (Y.W.); xiaozhiqiang@hnu.edu.cn (Z.X.); 18861822835@163.com (S.Z.); tanxinjia0209@163.com (X.T.); zhaoyifei@hnu.edu.cn (Y.Z.); liujmax2019@163.com (J.L.); 2 Agriculture Product Processing Institute, Hunan Academy of Agricultural Sciences, Changsha 410125, China; jiangning0806@outlook.com; 3 Hunan Key Lab of Fruits & Vegetables Storage, Processing, Quality and Safety, Hunan Agricultural Products Processing Institute, Changsha 410125, China; 4 Department of Life Sciences, Chalmers University of Technology, SE 412 96 Gothenburg, Sweden

**Keywords:** isoflavones, genistein, heme, malonyl-CoA, glycosylation

## Abstract

Isoflavones are predominantly found in legumes and play roles in plant defense and prevention of estrogen-related diseases. Genistein is an important isoflavone backbone with various biological activities. In this paper, we describe how a cell factory that can de novo synthesize genistein was constructed in *Saccharomyces cerevisiae*. Different combinations of isoflavone synthase, cytochrome P450 reductase, and 2-hydroxyisoflavone dehydratase were tested, followed by pathway multicopy integration, to stably de novo synthesize genistein. The catalytic activity of isoflavone synthase was enhanced by heme supply and an increased intracellular NADPH/NADP^+^ ratio. Redistribution of the malonyl-CoA flow and balance of metabolic fluxes were achieved by adjusting the fatty acid synthesis pathway, yielding 23.33 mg/L genistein. Finally, isoflavone glycosyltransferases were introduced into *S. cerevisiae*, and the optimized strain produced 15.80 mg/L of genistin or 10.03 mg/L of genistein-8-*C*-glucoside. This is the first de novo synthesis of genistein-8-*C*-glucoside in *S. cerevisiae*, which is advantageous for the green industrial production of isoflavone compounds.

## 1. Introduction

Flavonoids are a large family of polyphenols that are widely found in vegetables and fruits and are, therefore, commonly found in the human diet [1]. In recent years, there has been a keen interest in exploiting the bioactivity of flavonoids and their widespread application in the food and pharmaceutical industries [2]. In 2020, the global market for flavonoids was valued at USD 1497.7 million and is expected to reach USD 2717.8 million in ten years [3]. Isoflavones are important flavonoids that play an essential role in the treatment of women’s health issues [4], multiple sclerosis [5], and diabetes [6]. Genistein, an important isoflavonoid, was first identified in *Genista tinctoria* L. [7]. It is widespread in the legume family and called a phytoestrogen because of its structural similarity to human estrogen [8]. Genistein has antimicrobial properties and plays an important role in plant defense [9]. Regarding human diseases, research on genistein has focused on the prevention or mitigation of female-related [10] and cardiovascular diseases [11]. Many studies have demonstrated that the structural modification of genistein can result in better biological activities [12], such as glycosylated genistein derivatives, which have important potential for pharmaceutical applications [13,14].

In plants, isoflavone biosynthesis is based on naringenin [15]. Plants can use the tyrosine or phenylalanine pathway [16] to convert these amino acids to *p*-coumaric acid via phenylalanine ammonia lyase (PAL) and cinnamic acid 4-hydroxylase (C4H). Biosynthesis of naringenin from *p*-coumaric acid is catalyzed by 4-coumaric acid: coenzyme A ligase (4CL), chalcone synthase (CHS), and chalcone isomerase (CHI) [17]. The conversion of flavonoids to isoflavones requires the involvement of isoflavone synthase (IFS) [18]. IFS, a cytochrome P450 monooxygenase, catalyzes the production of the intermediate, 2-hydroxyisoflavone, from naringenin [19]. Expression of P450 enzymes is often coupled with cytochrome P450 reductase (CPR), making them more catalytically efficient [20]. Subsequently, 2-hydroxyisoflavone dehydratase (HID) dehydrates 2-hydroxyisoflavone to produce genistein [21]. Isoflavones are often found in plants as *O*- or *C*-glycosides [22] that are advantageous in terms of solubility, stability, and bioavailability [14]. Genistein is modified by 7-*O*-glycosylation to produce genistin, which is more bioavailable and readily absorbed in the intestine [23]. Flavonoid-*C*-glycosides have been less studied than *O*-glycoside compounds because of the low content of *C*-glycoside compounds in plants, and the mechanism of the *C*-glycosylation reaction has yet to be investigated [24]. A new *C*-glucosyltransferase (PlUGT43) with strong substrate specificity for genistein was reported to catalyze the conversion of genistein to genistein-8-*C*-glucoside [25].

Currently, the mainstream production of genistein relies on plant extraction and chemical synthesis [26]. Traditional methods have potential risks and pitfalls, such as using nonrenewable raw materials, low product yields, poor economics, a large consumption of toxic reagents, and waste emissions [27]. Microbial production has the advantages of strict regional stereo-and chemoselectivity, specific product configuration, and mild and environmentally friendly reactions; and the artificial recombination of synthetic pathways have the potential to replace traditional production technologies [28,29]. Kim et al. verified the feasibility of synthesizing genistein in *Escherichia coli* by removing the membrane-bound structural domains of IFS and CPR, accompanied by the addition of naringenin [30]. Recently, Liu et al. achieved de novo synthesis of genistein in a mixture of glucose and glycerol using a coculture system in *E. coli* [31]. *Saccharomyces cerevisiae* has an inherent advantage in the expression of P450 enzymes [32] and is more suitable as a chassis cell for constructing metabolic pathways involving them. Meng et al. made modular engineering modifications to *S. cerevisiae* for de novo genistein synthesis via sucrose fermentation [33]. Genistein is the basic skeleton of isoflavones, and many high value-added isoflavone derivatives can be obtained by its structural modification (e.g., glycosylation, alkylation, and hydroxylation) [34]. To produce genistin with glycerol, genistein is glycosylated in *E. coli* [35].

**Figure 1:**
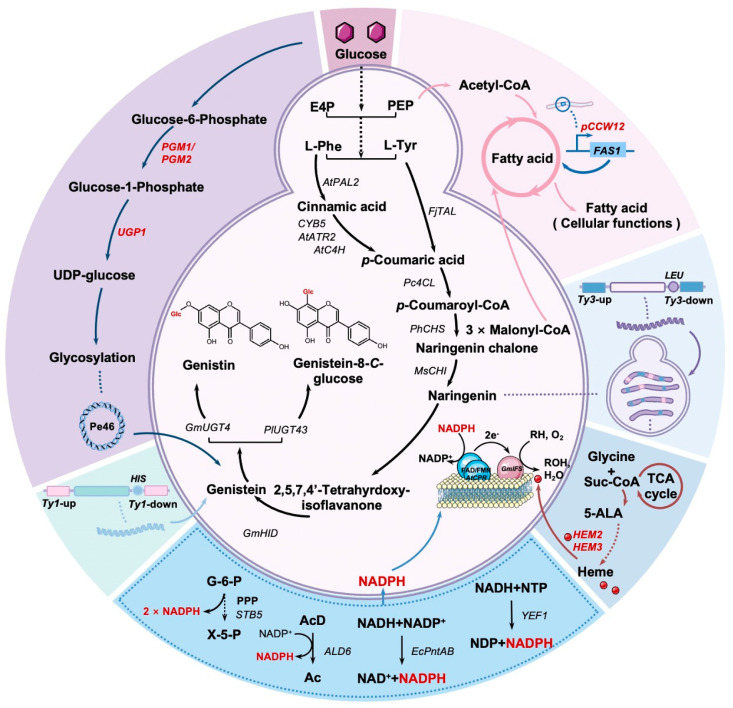
Systematic engineering for the de novo biosynthesis of genistein and glycosylated derivatives. The black lines indicate the biosynthetic pathways of the two isoflavone glycosides. The outermost six modules demonstrate the strategies in this paper. They are represented sequentially as a regulation of fatty acid metabolic pathways, multicopy site integration of the naringenin pathway, heme supply, intracellular NADPH production, multicopy site integration of the genistein pathway, and UDPG supply (clockwise). Notes on abbreviations: E4P, erythrose-4-phosphate; PEP, phosphoenolpyruvate; _L_-Phe, _L_-phenylalanine; _L_-Tyr, _L_-tyrosine; AtPAL2, phenylalanine ammonialyase from *Arabidopsis thaliana*; CYB5, yeast native cytochrome b5; AtATR2, cytochrome P450 reductase from *A. thaliana*; AtC4H, cinnamic acid-4-hydroxylase from *A. thaliana*; FjTAL, transaldolase from *Flavobacterium johnsoniae*; Pc4CL, 4-coumarate-coenzyme A ligase from *Petroselinum crispum*; PhCHS, chalcone synthase from *Petunia hybrida*; MsCHI, chalcone isomerase from *Medicago sativa*; AtCPR, cytochrome P450 reductase from *A. thaliana*; GmIFS, isoflavone synthase from *Glycine max*; GmHID, 2-hydroxyisoflavanone dehydratase from *G. max*; GmUGT4, isoflavone-7-*O*-glucosyltransferase from *G. max*; PlUGT43, isoflavone-8-*C*-glucosyltransferase from *Pueraria lobate*; FAS1, β-subunit of yeast fatty acid synthetase; Suc-CoA, succinyl-CoA; 5-ALA, 5-aminolevulinic acid; HEM2, 5-aminolevulinic acid dehydratase; HEM3, 4-porphobilinogen deaminase; G-6-P, glucose-6-phosphate; STB5, yeast native transcriptional factor; ALD6, cytoplasmic NADP^+^-dependent aldehyde dehydrogenase; EcPntAB, membrane-bound transhydrogenase from *E. coli*; YEF1, ATP-NADH kinase; HIS, histidine; PGM1/2, phosphoglucomutase 1/2; UGP1, UDP-glucose pyrophosphorylase; UDP-glucose, uridine diphosphate-glucose.

In the current study, we aimed to construct a genistein isoflavone scaffold platform to synthesize two isoflavone glycosides in *S. cerevisiae* (Figure 1). To construct and optimize the genistein pathway, different combinations of IFS, CPR, and HID were used for genistein biosynthesis. Next, we aimed to construct a strain that could de novo synthesize genistein based on multicopy integration. Optimization of the heme pathway and electron transport chain improved the catalytic efficiency of IFS. The metabolic flux in the competitive pathway was redistributed by fine-tuning the expression levels of related genes. We also introduced two separate glycosyltransferases while optimizing the supply of UDP-glucose (UDPG) to convert genistein into glycosylated derivatives with higher biological activity. Our study is the first to de novo synthesize genistein-8-*C*-glucoside in *S. cerevisiae.* The platform for *S. cerevisiae* genistein synthesis creates more possibilities for the microbial fermentation synthesis of other isoflavonoids. This study could provide new ideas for the green production of isoflavonoids.

## 2. Materials and Methods

### 2.1. Plasmids, Strain, and Reagents

Plasmids were constructed in *E. coli* JM109. The strains used in this study are listed in Table S1. All the genes used in this study and their source are listed in Table S2. All primers used in this study are listed in Table S3 and were synthesized by Sangon Bio (Shanghai, China). Phanta Max Super-Fidelity DNA Polymerase and the ClonExpress MultiS One-Step Cloning Kit were purchased from Vazyme Bio (Nanjing, China). Exogenous genes used in this study were codon-optimized and chemically synthesized by Sangon Bio (Shanghai, China), as shown in Table S4. The sites used in the study and their guide RNA sequences are listed in Table S5. All plasmids used in the study are listed in Table S6. Genistein, genistin, and genistein-8-*C*-glucoside standards were purchased from Yuanye Bio (Shanghai, China). The 20× yeast nitrogen base (YNB) medium was purchased from Sangon Bio (Shanghai, China).

### 2.2. Strain Cultivation

Luria–Bertani medium consisting of 10 g/L peptone, 5 g/L yeast extract, and 10 g/L NaCl was supplemented with 50 μg/mL ampicillin for the cultivation and screening of *E. coli*. Yeast extract peptone dextrose (YPD) medium composed of 20 g/L peptone, 10 g/L yeast extract, and 20 g/L glucose was used for *S. cerevisiae* seeding solution incubation and fermentation. YNB medium was used to screen the transformed yeast by adding 50 mg/L uracil, 50 mg/L leucine, 50 mg/L histidine, or 50 mg/L tryptophan to the YNB master batch, and 20 g/L glucose, as desired. Moreover, 1 g/L 5-fluorouracil (YPD + 5-FOA) was added to the YPD solid medium to exclude the URA3 marker for the selection of yeast cultures. The precultured seed solutions were transferred to 250 mL conical flasks containing 25 mL YPD medium at 1% (*v*/*v*) inoculum and further cultured at 30 °C with 220 rpm. Fermentation samples were collected at 72 h and 96 h for analysis.

### 2.3. Genetic Manipulation

All the *S. cerevisiae* strains constructed in this study were derived from the genetic background of *S. cerevisiae* CEN.PK2-1D-derivative C800 (*MATα*; *ura3-52*; *trp1-289; leu2-3,112*; *his3Δ1*; *MAL2-8C*; *SUC2*; *gal80::G418*) and are detailed in Table S1. All the *S. cerevisiae* endogenous promoters, terminators, and genes used in this study were amplified with C800 genomic DNA as a template. All fragments were ligated into plasmids using the ClonExpress MultiS One-Step Cloning Kit (Vazyme Bio, Nanjing, China), and fusion of fragments performed using fusion PCR. In this study, the CRISPR/Cas9 system was used for gene overexpression and knockdown, and the DNA fragments constructed via PCR assembly integrated into the target loci of the genome. Guide RNAs were designed online (http://chopchop.cbu.uib.no/, accessed on 29 January 2024). Transformation of *S. cerevisiae* was performed using the lithium acetate method.

### 2.4. Analytical Methods

Fermentation broths (500 μL) were collected for high-performance liquid chromatography (HPLC) analysis. A methanol solution was used to extract genistein from the cells. An equal volume of methanol solution was added to the yeast fermentation broth, and the cells resuspended through vortexing for 5 min. The obtained mixture was centrifuged at 12,000× *g* for 2 min, and the supernatant was filtered through a 0.22 μm filter membrane. HPLC analysis for quantification of the supernatant was performed using a Prominence LC-20A instrument (Shimadzu, Kyoto, Japan) equipped with a diode array detector and an inert-sustained C18 250 mm × 4.6 mm column (particle size 5 μm; Shimadzu).

The absorbances of *p*-coumaric acid, naringenin, genistein, genistin, and genistein-8-*C*-glucoside in the fermentation broth were measured at 284 nm. The mobile phase comprised 0.1% aqueous formic acid solution (A) and 100% acetonitrile (B) at a flow rate of 1 mL/min. HPLC program mode: 0 min, 5% B; 15 min, 20% B; 30 min, 25% B; 35 min, 30% B; 40 min, 30% B; 60 min, 40% B; 64 min, 5% B; 65 min, 5% B. The column oven temperature was maintained at 30 °C, and 10 µL of each sample was used for analysis.

## 3. Results

### 3.1. Construction of the Genistein Synthesis Pathway

**Figure 2:**
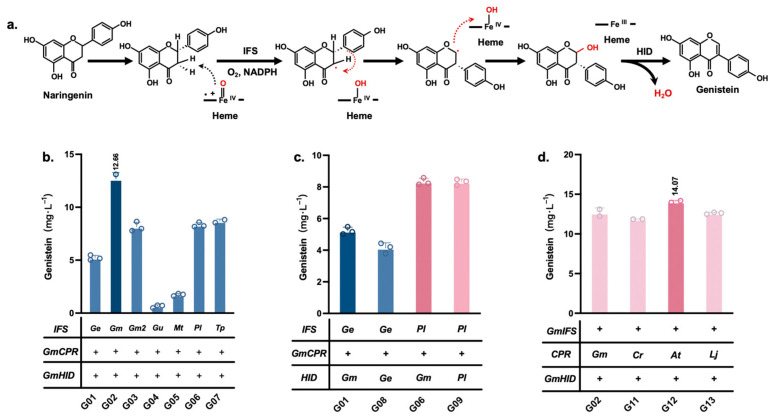
Construction of a synthetic plate for genistein. (**a**) The mechanism of naringenin-to-genistein conversion catalyzed by IFS and HID. (**b**) To screen the effect of different IFS on genistein synthesis with 400 mg/L naringenin addition. (**c**) Comparison of the effects on genistein synthesis by different HIDs expression with 400 mg/L naringenin addition. (**d**) Effect of different CPRs on genistein synthesis with 400 mg/L naringenin addition. Data are presented as mean ± SD.

Biosynthesis of genistein from naringenin requires the involvement of IFS and HID [18]. IFS catalyzes the B-ring aryl migration of naringenin, which is essential for the construction of the isoflavone pathway (Figure 2a). Therefore, we examined IFSs from different plant sources, including *Glycyrrhiza echinata*, *Glycine max*, *Glycyrrhiza uralensis*, *Medicago truncatula*, *Pueraria lobata*, and *Trifolium patense*. These IFSs were combined with CPR and HID, both of which were from *Glycine max*, and coexpressed in strain C800. Genistein was detected in all constructed strains (G01–07) with the addition of 400 mg/L naringenin. G02 coexpressing a combination of *GmIFS*, *GmCPR*, and *GmHID* accumulated the highest level of genistein (12.66 mg/L) (Figure 2b).

To verify the effects of different sources of HIDs on genistein synthesis, strain G08 was obtained by replacing *GmHID* with *GeHID* in strain G01, and strain G09 was obtained by replacing *GmHID* with *PlHID* in strain G06. The results showed that *GmHID* exhibited the best catalytic activity (Figure 2c). The effects of *CPRs* from different plant sources on genistein synthesis were also explored. We separately coexpressed *GmIFS* and *GmHID* with *CPRs* from three different sources (*Catharanthus roseus*, *Arabidopsis thaliana*, and *Lotus japonicus*) and constructed strains G11–13. The highest genistein titer obtained was 14.07 mg/L in G12, with 400 mg/L naringenin addition (Figure 2d). Therefore, *GmIFS*, *AtCPR*, and *GmHID* from G12 were used to construct the subsequent de novo genistein-synthesizing strains.

### 3.2. Stable De Novo Synthesis of Genistein via Double Multicopy Pathway Integration

**Figure 3:**
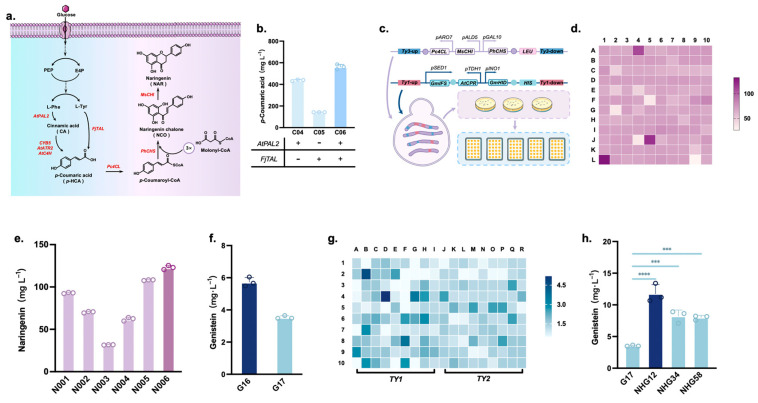
Building the de novo biosynthetic pathway for genistein. (**a**) Naringenin biosynthetic pathway. (**b**) Effect of expression of different genes on *p*-coumaric acid synthesis. (**c**) Schematic of multicopy site integration in the naringenin and genistein synthesis pathways. (**d**) Analysis of multicopy site integration of the naringenin synthesis pathway. (**e**) Shake flask rescreen fermentation by multiple-copy site-integrated strains of the naringenin pathway. (**f**) Comparison of differences between plasmid expression and single-copy integrated expression of the genistein synthesis pathway. (**g**) Analysis on the integration of the genistein synthesis pathway at the *Ty1* or *Ty2* transposons in *S. cerevisiae*. (**h**) Shake flask rescreen fermentation by multiple-copy site-integrated strains of the genistein pathway. Asterisks denote the statistical significance of a two-tailed *t*-test. *** *p* < 0.001, **** *p* < 0.0001. Data are presented as mean ± SD.

To achieve de novo genistein synthesis, it was first necessary to construct a naringenin-producing strain (Figure 3a). Initially, genes involved in the PAL and TAL pathways were integrated into the genome of strain C800, including phenylalanine ammonia-lyase (PAL2), C4H, and acytochrome P450 reductase (ATR2), which are all from *A. thaliana*, cytochrome B5 (CYB5) from *S. cerevisiae*, and TAL from *Flavobacterium johnsoniae*, to obtain strain C06. This strain could synthesize 561.6 mg/L *p*-coumaric acid at 72 h (Figure 3b). Next, the naringenin synthesis pathway genes, *Pc4CL* (*Petroselinum crispum*), *PhCHS* (*Petunia hybrida*), and *MsCHI* (*Medicago sativa*), were integrated into the multicopy locus, *Ty3*, of C06 (Figure 3c), and 120 strains with different copy numbers were constructed. Among these, six strains (N001-6) with the highest naringenin titers were obtained in 24-well plates (Figure 3d). Finally, the six strains were rescreened using shake flask fermentations, where strain N006 showed the highest naringenin production at 122.9 mg/L (Figure 3e). Therefore, strain N006 was selected as the chassis for genistein production.

The best combination selected for the genistein synthesis pathway (*GmIFS*, *AtCPR*, and *GmHID*) was expressed as a plasmid in N006 to obtain strain G16, which produced 5.73 mg/L genistein. To facilitate subsequent pathway optimization, we integrated this combination into the *EXG1* locus of the N006 genome to obtain strain G17. The result showed that the G17 strain could only produce 3.54 mg/L genistein, which was lower than that of G16 (Figure 3f). It is hypothesized that genistein synthesis may be limited by gene copy number. Therefore, the genistein pathway was integrated into the multicopy sites, *Ty1* or *Ty2*, in N006, and 180 strains screened for fermentation in 24-well plates (Figure 3g). Three strains (NHG12, NHG34, and NHG58) were evaluated for their ability to produce high genistein titers in shake flasks, and the results showed that the genistein titer of NHG12 could reach 11.75 mg/L. Its titer increased by 309.4% compared with that of G17 (Figure 3h). Thus, the copy number of key genes is one of the rate-limiting factors in genistein synthesis.

### 3.3. Enhancement of Isoflavone Synthase Catalytic Efficiency and Yeast Growth-Promoting Modification

**Figure 4:**
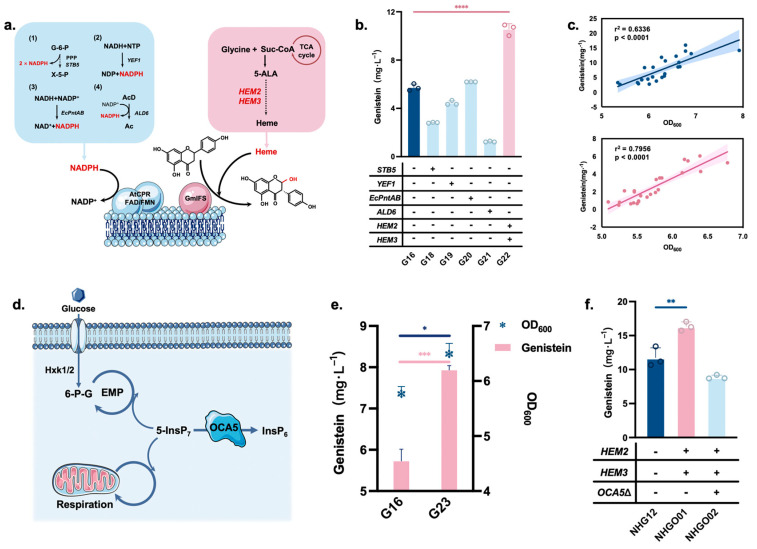
Enhancement of IFS catalytic efficiency and yeast growth-promoting modification. (**a**) Schematic diagram of the strategy for NADPH production and heme supply. (**b**) Effect of NADPH production and heme supply on genistein titer. (**c**) A positive correlation between OD_600_ and genistein titer. Blue samples indicate the relationship between genistein titers and OD_600_ for strains fermented with 400 mg/L naringenin addition. r^2^ = 0.6336, *p* < 0.0001. Pink samples indicate the relationship between titers and OD_600_ for the de novo synthesis of genistein by fermentation using glucose as the sole carbon source. r^2^ = 0.7956, *p* < 0.0001. (**d**) Mechanism of OCA5 action in the intracellular compartment. Hxk1/2, hexokinase; 5-InsP7, 5-diphosphoinositol 1,2,3,4,6-pentakisphosphate; InsP6, inositol hexakisphosphate. (**e**) Effect of *OCA5* deletion on genistein synthesis and OD_600_. (**f**) Effective strategies to integrate into the genome of NHG12. Asterisks denote the statistical significance of a two-tailed *t*-test. * *p*  < 0.05, ** *p* < 0.01, *** *p* < 0.001, **** *p* < 0.0001. Data are presented as mean ± SD.

In addition to the impact of copy number, other factors that influence genistein synthesis were also investigated in G16. NADPH regeneration and intracellular heme supply were optimized to enhance IFS enzyme activity (Figure 4a). *STB5*, *YEF1*, *EcPntAB*, and *ALD6* were overexpressed in strain G16 to enhance NADPH regeneration, producing strains G18–21. To alleviate the limitations of heme synthesis, *HEM2* and *HEM3* were overexpressed in strain G16 to obtain strain G22. The results showed that the heme supply strategy (G22) significantly improved the catalytic efficiency of IFS (Figure 4b). The strain accumulated 10.55 mg/L genistein, an 84.1% increase over that produced in G16. During the experiment, we found a positive correlation between the genistein titer and strain growth (Figure 4c). The deletion of *OCA5* resulted in central carbon metabolism homeostasis in *S. cerevisiae* (Figure 4d), improved the efficiency of cellular energy metabolism, and led to more rapid cell growth [36]. Therefore, *OCA5* was knocked out in the G16 and strain G23 was obtained. The optical density of G23 measured at 600 nm increased by 19.1% compared with that of the parent strain, and genistein accumulation increased by 38.6% (7.94 mg/L) (Figure 4e).

As enhanced heme supply significantly increased the IFS catalytic efficiency, *HEM2* and *HEM3* were integrated into the *EXG1* genome of NHG12 to obtain strain NHGO01. NHGO01 was able to synthesize 16.35 mg/L genistein, which was a 39.1% increase over that in NHG12 (Figure 4f). As the *OCA5* deletion promoted strain growth and favored genistein synthesis in G23, strain NHGO02 was obtained by knocking down *OCA5* in NHGO01. However, the genistein titer of this strain decreased to 8.88 mg/L (Figure 4f). This could be due to the *OCA5* deletion decreasing intracellular NADPH/NADP^+^ levels [36].

### 3.4. Improved Conversion of Malonyl-CoA to Genistein

**Figure 5:**
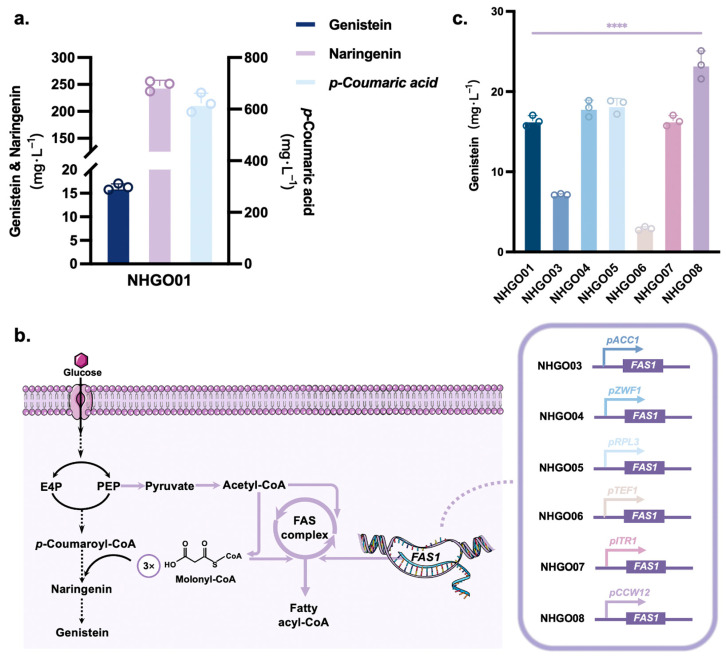
Optimization of the malonyl-CoA supply. (**a**) Demonstration of titers in NHGO01 for genistein, naringenin, and *p*-coumaric acid. (**b**) Schematic diagram of *FAS1,* which regulates the fatty acid synthesis pathway, to redistribute the malonyl-CoA flow. (**c**) Effect of different promoters for *FAS1* expression on genistein titers. Asterisks denote the statistical significance of a two-tailed *t*-test. **** *p* < 0.0001. Data are presented as mean ± SD.

A low malonyl coenzyme A (malonyl-CoA) content is one of the factors limiting the conversion of *p*-coumaric acid to naringenin. The NHGO01 strain showed high accumulation of *p*-coumaric acid during genistein synthesis (Figure 5a). It has been speculated that there may be insufficient levels of malonyl-CoA, where cytoplasmic malonyl-CoA is mostly used for fatty acid synthesis [37]. Fatty acid synthesis in *S. cerevisiae* is associated with a *FAS1* and *FAS2* complex, and the product of *FAS1* promotes the expression of *FAS2* [38]. Fine-tuning *FAS1* expression could redistribute malonyl-CoA metabolic flow and decrease fatty acid synthesis to increase the naringenin titer (Figure 5b). We selected a set of *S. cerevisiae* promoters (*ACC1p*, *ZWF1p*, *RPL3p*, *TEF1p*, *ITP1p*, and *CCW12p*) to individually regulate *FAS1* expression and obtained seven strains (NHGO03-08) where the strong promoter *TEF1p* was used as a control. The results showed that the NHGO08 strain with *FAS1* regulated by *CCW12p* had the highest genistein titer, which was 42.69% higher than that obtained by NHGO01 (Figure 5c).

### 3.5. Production of Glycosylated Derivatives of Genistein

**Figure 6:**
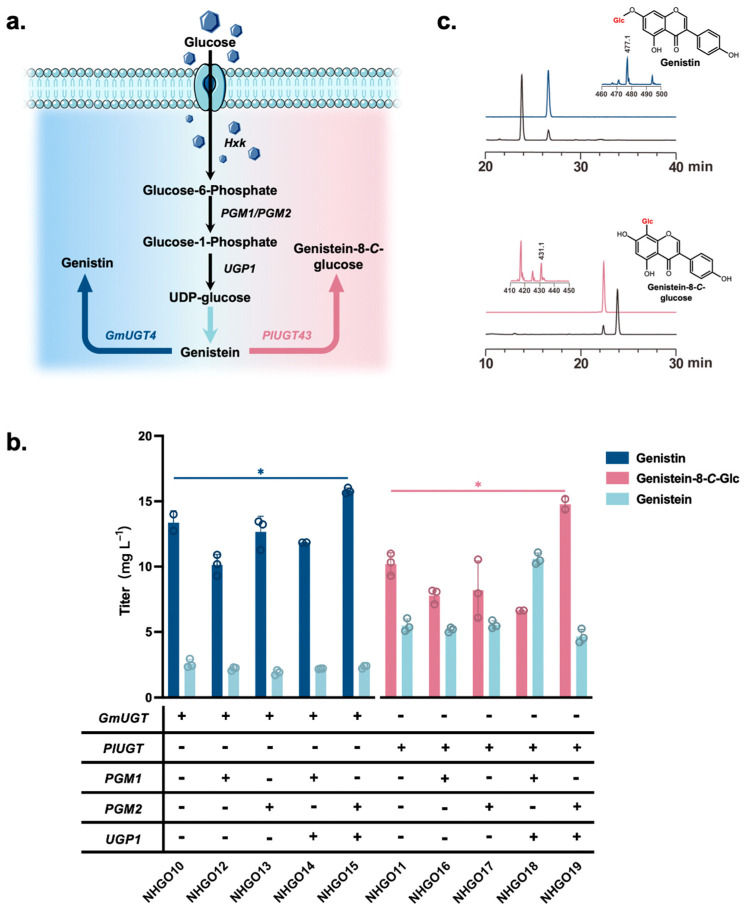
Synthesis of glycosylated derivatives of genistein. (**a**) Demonstration of the isoflavone glycoside synthesis pathway and the principle of glycosylation optimization strategy. (**b**) Effect of the expression of two glycosyltransferases, *PGM1*, *PGM2*, and *UGP1* on the synthesis of isoflavone glycosides. (**c**) LC-MS for genistein and genistein-8-*C*-glucoside. Asterisks denote the statistical significance of a two-tailed *t*-test. * *p* <  0.05. Data are presented as mean ± SD.

The biosynthesis of flavonoid glycosides is catalyzed by glucosyltransferases and mediated by UDP sugars [22]. Genistein was converted to genistin and genistein-8-*C*-glucoside, catalyzed, respectively, by *GmUGT4* and *PlUGT43* with the glycosyl donor, UDPG (Figure 6a). These two glycosyltransferases were separately overexpressed in NHGO08 to obtain the strains NHGO10–11. As a result, NHGO10 produced genistin (13.36 mg/L) and NHGO11 produced genistein-8-*C*-glucoside (7.65 mg/L) (Figure 6b). The production of genistin and genistein-8-*C*-glucoside was further verified using liquid chromatography–mass spectrometry (Figure 6c). UDPG production by *S. cerevisiae* requires the expression of phosphorylase (PGM1/PGM2) and glucose-1-uridyltransferase (UGP1) [22]. Strain NHGO12-19 was constructed by expressing *PGM1/PGM2* alone or in combination with *UGP1* in NHGO09-10. The results showed that the NHGO15 and the NHGO19 strains overexpressing *PGM2* and *UGP1* could produce 15.80 mg/L genistin and 10.03 mg/L genistein-8-*C*-glucoside. In the synthesis of isoflavone glycosides, UDPG supply increased the titer by 18.26% and 31.11%, respectively, compared with that when glycosyltransferase was expressed alone.

## 4. Discussion

Isoflavones have unlimited potential for research and applications in pharmaceuticals, plant pest, and disease defense research fields [6]. Genistein, the basic skeleton of isoflavones, is widely recognized for its antioxidant and anticancer functions [10]. Microbial synthesis technology is developing rapidly, with the possibility of replacing traditional processes, and have better adaptability to the new international competitive environment [28]. In this study, we constructed a cell factory in *S*. *cerevisiae* for the production of genistein and its glycoside derivatives (Figure 1). We initially explored construction of the genistein pathway through screening enzymes involved in its synthesis. The naringenin and genistein synthesis pathways were then integrated into multicopy sites of the yeast genome, which enabled the stable de novo synthesis of genistein. After optimizing the metabolic flux, cell growth, and activity of the key enzyme, IFS, an increased genistein titer of 23.33 mg/L was obtained. Finally, glycosyltransferases (GmUGT4 [39] and PlUGT43 [25]) were introduced to convert genistein to genistin and genistein-8-*C*-glucoside.

IFS is a member of the P450s family and mediates a major rate-limiting step in genistein synthesis [18]. The kernel of the IFS reaction is oxygen catabolism and substrate monooxygenation, which are accomplished by the flavin adenine dinucleotide/flavin-containing mononucleotide attached to the CPR to transfer electrons from NADP(H) to the heme iron center (Figure 4a) [40]. Thus, enhancement of NADPH and heme synthesis can increase the IFS-regulated conversion efficiency of naringenin. Four scenarios were designed to optimize the pathway that promotes NADPH production (Figure 4a): (1) introduction of the transcription factor, *Stb5*. In *S. cerevisiae*, the pentose phosphate pathway (PPP) is the major NADPH synthesis pathway involved in cellular metabolism, which *Stb5* can activate [41]. (2) Overexpression of *YEF1*, an ATP-NADH kinase that enables NADH phosphorylation, thereby increasing the concentration of electron sources [42]. (3) Overexpression of *EcPntAB*, a transhydrogenase from *E. coli* that enables the reduction in NADP+ [43]. (4) Overexpression of *ALD6*, an aldehyde dehydrogenase encoded by *ALD6* that converts acetaldehyde to acetate and promotes NADPH production [44]. The 5-aminolevulinic acid dehydratase encoded by *HEM2* and 4-bilirubinogen deaminase encoded by *HEM3* in *S. cerevisiae* regulate rate-limiting steps in the heme synthesis pathway (Figure 4a), and overexpression of these two genes was used for heme supply [45]. Results showed that the heme supply in these strategies significantly increased genistein synthesis (Figure 4f), which reflected a more pronounced effect of heme concentration on IFS activity.

As we found a positive correlation between genistein and optical density values measured at 600 nm (Figure 4c), improving the growth of *S. cerevisiae* may promote genistein production. Inositol pyrophosphatase, encoded by *OCA5* in *S. cerevisiae*, regulates cellular glycolytic pathways and respiration [36]. This enzyme could be regulated by modulating the level of 5-diphospho-1,2,3,4,6-pentakisphosphate (5-InsP7) (Figure 4d). The *OCA5* knockout promoted both cell growth and genistein synthesis in strain G23 (Figure 4e). When *OCA5* was knocked out in the NHGO02 strain, we found that the genistein titer was low (Figure 4f). This may be because the *OCA5* deletion drives cellular energy metabolism to promote growth while decreasing the intracellular NADPH/NADP+ concentration, which negatively affects the P450 enzyme system [36].

The increase in malonyl-CoA levels via modulation of the fatty acid synthesis pathway favored the conversion of *p*-coumaric acid to naringenin, which in turn promoted genistein synthesis (Figure 5b). Therefore, the supply of malonyl-CoA, which is converted into *p*-coumaroyl-CoA with *p*-coumaric acid, is the main limiting factor [46]. Malonyl-CoA is used to supply cellular fatty acids and maintain cellular function, in addition to participating in naringenin synthesis [37]. Yeast cell fatty acid synthase consists of FAS1 (*β*-subunit) and FAS2 (*α*-subunit), and increased levels of FAS1 protein stimulate *FAS2* gene expression [37,38]. We regulated *FAS1* expression by replacing it with a weaker promoter to balance the malonyl-CoA diversion ratio. It was found that the replacement of *CCW12p* with *FAS1p* increased the genistein titer by 98.6% in strain NHGO08 over that in NHGO01 (Figure 5c). The final genistein titer obtained was 23.33 mg/L, when glucose was used as the sole carbon source.

Optimization of UDPG supply is a commonly used tool in glycosylation pathway construction [47]. In *S. cerevisiae*, glucose is first converted to glucose 6-phosphate (G-6-P), then to glucose 1-phosphate (G-1-P) by the action of *PGM1/PGM2*, and UDPG is finally catalyzed by *UGP1* [22]. These genes were overexpressed to expand the UDPG pool and overcome the rate-limiting challenges in the glycoside product synthesis pathway (Figure 6a). The expression of different glycosyltransferases produced 15.80 mg/L genistin (NHGO15) and 10.03 mg/L genistein-8-*C*-glucoside (NHGO19) (Figure 6b). Our study achieved the first de novo synthesis of genistin and genistein-8-*C*-glucoside in *S*. *cerevisiae*, providing a reference for future isoflavone research. Further improvements in enzyme engineering, subcellular organelle localization, and strain-directed domestication could drive the synthesis of other isoflavones.

## Data Availability

Data are contained within the article and Supplementary Materials.

## Supplementary Materials

The following supporting information can be downloaded at: https://www.mdpi.com/article/10.3390/jof10030176/s1, Table S1: Strains used in this study; Table S2: Genes used in the present study; Table S3: Primers used in the present study; Table S4: Codon optimized exogenous gene sequences; Table S5: Guide RNA sequences used in this study; Table S6: Plasmids used in this study.
