# Supplementary material for: Systematic Engineering of Saccharomyces cerevisiae for the De Novo Biosynthesis of Genistein and Glycosylation Derivatives
Source: J Fungi (Basel). 2024 Feb 26;10(3):176. doi: 10.3390/jof10030176 (PMC10970955; doi:10.3390/jof10030176)
Supplement: Supplementary file 1 [file jof-10-00176-s001.zip › jof-2823599-supplementary.pdf]

Supplementary Information

Systematic Engineering of *Saccharomyces cerevisiae* for the De Novo Biosynthesis of Genistein and Glycosylation Derivatives

Yongtong Wang [1,2,3], Zhiqiang Xiao [1,2,3], Siqi Zhang [1,2,3], Xinjia Tan [1,2,3], Yifei Zhao [1,2,3], Juan Liu [1,2,3,4], Ning Jiang [2,3] and Yang Shan [1,2,3,*]

1 Longping Branch, College of Biology, Hunan University, Changsha 410125, China; quarknorthstar@163.com (Y.W.); xiaozhiqiang@hnu.edu.cn (Z.X.); 18861822835@163.com (S.Z.); tanxinjia0209@163.com (X.T.); zhaoyifei@hnu.edu.cn (Y.Z.); liujmax2019@163.com (J.L.)
2 Agriculture Product Processing Institute, Hunan Academy of Agricultural Sciences, Changsha 410125, China; jiangning0806@outlook.com
3 Hunan Key Lab of Fruits & Vegetables Storage, Processing, Quality and Safety, Hunan Agricultural Products Processing Institute, Changsha 410125, China
4 Department of Life Sciences, Chalmers University of Technology, SE 412 96 Gothenburg, Sweden
* Correspondence: sy6302@sohu.com; Tel.: +86-0731-84691289; Fax: +86-0731-82873368

Table S1 Strains used in this study.

Strains	Parent strain	Description	Sources
S. cerevisiae CENPK2-1D	CENPK2-1D	*MATα; ura3-52; trp1-289; leu2-3,112; his3Δ1; MAL2-8C; SUC2*	Laboratory
S. cerevisiae C800	CENPK2-1D	*gal80::G418, CENPK2-1D*	Laboratory
C04	C800	*XI-5::(GAL10p-AtC4H-CYCIt)+(GAL10p-AtPAL2-CIT2t)+(THD3p-AtATR2-SDH1t)+(SED1p-CYB5-PDB1t)*	This study
C05	C800	*XI-5::(GAL10p-AtC4H-CYCIt)+(THD3p-AtATR2-SDH1t)+(SED1p-CYB5-PDB1t); XI-2::(GAL7p-FjTAL-DIT1t)*	This study
C06	C800	*XI-5::(GAL10p-AtC4H-CYCIt)+(GAL10p-AtPAL2-CIT2t)+(THD3p-AtATR2-SDH1t)+(SED1p-CYB5-PDB1t); XI-2::(GAL7p-FjTAL-DIT1t)*	This study
N001	C06	*Ty3::GAL10p-PhCHS-ALD5p-MsCHI-ARO7p-Pc4CL-LEU2p-LEU2, C800*	This study
N002	C06	*Ty3::GAL10p-PhCHS-ALD5p-MsCHI-ARO7p-Pc4CL-LEU2p-LEU2, C800*	This study

N003	C06	*Ty3::GAL10p-PhCHS-ALD5p-MsCHI-ARO7p-Pc4CL-LEU2p-LEU2, C800*	This study
N004	C06	*Ty3::GAL10p-PhCHS-ALD5p-MsCHI-ARO7p-Pc4CL-LEU2p-LEU2, C800*	This study
N005	C06	*Ty3::GAL10p-PhCHS-ALD5p-MsCHI-ARO7p-Pc4CL-LEU2p-LEU2, C800*	This study
N006	C06	*Ty3::GAL10p-PhCHS-ALD5p-MsCHI-ARO7p-Pc4CL-LEU2p-LEU2, C800*	This study
G01	C800	*pY26-SED1p-GeHIS-Ter22-GmCPR-TDH1p-INO1p-GmHID-CYC1t*	This study
G02	C800	*pY26-SED1p-GmIFS-Ter22-GmCPR-TDH1p-INO1p-GmHID-CYC1t*	This study
G03	C800	*pY26-SED1p-GmIFS2-Ter22-GmCPR-TDH1p-INO1p-GmHID-CYC1t*	This study
G04	C800	*pY26-SED1p-GuIFS-Ter22-GmCPR-TDH1p-INO1p-GmHID-CYC1t*	This study
G05	C800	*pY26-SED1p-MtIFS-Ter22-GmCPR-TDH1p-INO1p-GmHID-CYC1t*	This study
G06	C800	*pY26-SED1p-PlIFS-Ter22-GmCPR-TDH1p-INO1p-GmHID-CYC1t*	This study
G07	C800	*pY26-SED1p-TpIFS-Ter22-GmCPR-TDH1p-INO1p-GmHID-CYC1t*	This study

G08	C800	*pY26-SED1p-GeHIS-Ter22-GmCPR-TDH1p-INO1p-GeHID-CYC1t*	This study
G09	C800	*pY26-SED1p-PlIFS-Ter22-GmCPR-TDH1p-INO1p-PlHID-CYC1t*	This study
G11	C800	*pY26-SED1p-GmIFS-Ter22-CrCPR2-TDH1p-INO1p-GmHID-CYC1t*	This study
G12	C800	*pY26-SED1p-GmIFS-Ter22-AtCPR-TDH1p-INO1p-GmHID-CYC1t*	This study
G13	C800	*pY26-SED1p-GmIFS-Ter22-LjCPR-TDH1p-INO1p-GmHID-CYC1t*	This study
G16	N006	*pY26-SED1p-GmIFS-Ter22-AtCPR-TDH1p-INO1p-GmHID-CYC1t*	This study
G17	N006	*EXG1::SED1p-GmIFS-Ter22-AtCPR-TDH1p-INO1p-GmHID-CYC1t;*	This study
G18	N006	*pY26-SED1p-GmIFS-Ter22-AtCPR-TDH1p-INO1p-GmHID-CYC1t; PRS424-SED1p-STB5-TEr22*	This study
G19	N006	*pY26-SED1p-GmIFS-Ter22-AtCPR-TDH1p-INO1p-GmHID-CYC1t; pRS424-SED1p-YEF1-TEr22*	This study
G20	N006	*pY26-SED1p-GmIFS-Ter22-AtCPR-TDH1p-INO1p-GmHID-CYC1t; pRS424-SED1p-EcPntAB-TEr22*	This study
G21	N006	*pY26-SED1p-GmIFS-Ter22-AtCPR-TDH1p-INO*	This

		1p-GmHID-CYC1t; *pRS424-SED1p-ALD6-TEr22*	study
G22	N006	*pY26-SED1p-GmIFS-Ter22-AtCPR-TDH1p-INO1p-GmHID-CYC1t;* *pRS424-SED1p-HEM2-TEr22-HEM3-TDH3p*	This study
G23	N006	*oca5Δ;* *pY26-SED1p-GmIFS-Ter22-AtCPR-TDH1p-INO1p-GmHID-CYC1t*	This study
NHG 12	GO06	*TY1::SED1p-GmIFS-Ter22-AtCPR-TDH1p-INO1p-GmHID-CYC1t*	This study
NHG 34	GO06	*TY1::SED1p-GmIFS-Ter22-AtCPR-TDH1p-INO1p-GmHID-CYC1t*	This study
NHG 58	GO06	*TY1::SED1p-GmIFS-Ter22-AtCPR-TDH1p-INO1p-GmHID-CYC1t*	This study
NHGO01	NHG12	*exg1::SED1p-HEM2-TEr22-HEM3-TDH3p*	This study
NHGO02	NHGO01	*oca5Δ,*	This study
NHGO03	NHGO01	*fas1::(ACC1p-FAS1)*	This study
NHGO04	NHGO01	*fas1::(ZWF1p-FAS1)*	This study

NHGO05	NHGO01	*fas1::(RPL3p-FAS1)*	This study
NHGO06	NHGO01	*fas1::(TEF1p-FAS1)*	This study
NHGO07	NHGO01	*fas1::(ITR1p-FAS1)*	This study
NHGO08	NHGO01	*fas1::(CCW12p-FAS1)*	This study
NHGO10	NHGO08	*pY26-SED1p-GmUGT4-DIT1t*	This study
NHGO11	NHGO08	*pY26-SED1p-PlUGT43-DIT1t*	This study
NHGO12	NHGO08	*pY26-SED1p-GmUGT4-DIT1t; pRS424-TDH1p-PGM1-ADH1t*	This study
NHGO13	NHGO08	*pY26-SED1p-GmUGT4-DIT1t; pRS424-TDH1p-PGM2-ADH1t*	This study
NHGO14	NHGO08	*pY26-SED1p-GmUGT4-DIT1t; pRS424-TDH1p-PGM1-ADH1t-INO1p-UGP1-CYC1t*	This study
NHGO15	NHGO08	*pY26-SED1p-GmUGT4-DIT1t; pRS424-TDH1p-PGM2-ADH1t-INO1p-UGP1-CYC1t*	This study

NHGO16	NHGO08	*pY26-SED1p-PlUGT43-DIT1t;* *pRS424-TDH1p-PGM1-ADH1t*	This study
NHGO17	NHGO08	*pY26-SED1p-PlUGT43-DIT1t;* *pRS424-TDH1p-PGM2-ADH1t*	This study
NHGO18	NHGO08	*pY26-SED1p-PlUGT43-DIT1t;* *pRS424-TDH1p-PGM1-ADH1t-INO1p-UGP1-CYC1t*	This study
NHGO19	NHGO08	*pY26-SED1p-PlUGT43-DIT1t;* *pRS424-TDH1p-PGM2-ADH1t-INO1p-UGP1-CYC1t*	This study

Table S2 Genes used in the present study

Gene	Original organisms	EC numbers
AtC4H	*Arabidopsis thaliana*	1.14.14.91
AtPAL2	*Arabidopsis thaliana*	4.3.1.24
AtATR2	*Arabidopsis thaliana*	1.6.2.4
FjTAL	*Flavobacterium johnsoniae*	2.2.1.2
PhCHS	*Petunia hybrida*	2.3.1.74
MsCHI	*Medicago sativa*	5.5.1.6
Pc4CL	*Petroselinum crispum*	6.2.1.12
GeHIS	*Glycyrrhiza echinata*	1.14.14.87
GmIFS	*Glycine max*	1.14.14.87
GmIFS2	*Glycine max*	1.14.14.87
GuIFS	*Glycyrrhiza uralensis*	1.14.14.87
MtIFS	*Medicago truncatula*	1.14.14.87
PlIFS	*Pueraria lobata*	1.14.14.87
TpIFS	*Trifolium patense*	1.14.14.87
GmHID	*Glycine max*	4.2.1.105
GeHID	*Glycyrrhiza echinata*	4.2.1.105
PlHID	*Pueraria lobata*	4.2.1.105
GmCPR	*Glycine max*	1.6.2.4
CrCPR2	*Catharanthus roseus*	1.6.2.4

AtCPR	*Arabidopsis thaliana*	1.6.2.4
LjCPR	*Lotus japonicus*	1.6.2.4
EcPntAB	*Escherichia coli*	7.1.1.1
GmUGT4	*Glycine max*	2.4.1.170
PlUGT43	*Pueraria lobata*	2.4.1.17
STB5	*Saccharomyces cerevisiae*	7.3.2.1
YEF1	*Saccharomyces cerevisiae*	2.7.1.23
ALD6	*Saccharomyces cerevisiae*	1.2.1.3
HEM2	*Saccharomyces cerevisiae*	4.2.1.24
HEM3	*Saccharomyces cerevisiae*	2.5.1.61
PGM1	*Saccharomyces cerevisiae*	5.4.2.2
PGM2	*Saccharomyces cerevisiae*	5.4.2.7
UGP1	*Saccharomyces cerevisiae*	2.7.7.9
CYB5	*Saccharomyces cerevisiae*	1.6.2.2

Table S3 Primers used in the present study

Primers	Nucleotide sequence (5′ - 3′)	Application
**Construction of the synthesis pathway for p-coumaric acid**		
GAL10p-1-F	ACAACATGGCTTCAATTTGATCCATT ATAGTTTTTCTCCTTGACGTTAAAGT ATAG	Amplification of *GAL10p* promoter
GAL10p-1-R	AGACTTTTCCAACAACAACAAATCC ATTTATATTGAATTTTCAAAAATTCTT ACTTTTT	Amplification of *GAL10p* promoter
AtC4H-F	ATCGACAAAGGAAAAGGGGCCTGTT TAACAGTTTCTTGGCTTCATAACAAT AATAGAG	Amplification of *AtC4H* fragment
AtC4H-R	ATGGATTTGTTGTTGTTGGAAAAGTC TTTG	Amplification of *AtC4H* fragment
CYCIt-F	ACAGGCCCCTTTTCCTTTGTCGATA	Amplification of *CYC1t* terminator
CYCIt-R	GCAAATTAAAGCCTTCGAGCGTCC	Amplification of *CYC1t* terminator
AtPAL2-F	ATGGATCAAATTGAAGCCATGTTGTG TG	Amplification of *AtPAL2* fragment

AtPAL2-R	GTAAAAGTAGGATGTAATCCAAATTT AACAAATTGGAATTGGAGCACCG	Amplification of *AtPAL2* fragment
CIT2t-F	TTGGATTACATCCTACTTTTACACCC	Amplification of *CIC2t* terminator
CIT2t-R	GCCGGAATAGTGCAAATTGTATG	Amplification of *CIC2t* terminator
THD3p-F	AGCAATCAGTGGTGTATGGAAGGTA GAATAAAAAACACGCTTTTTCAGTTC GAG	Amplification of *TDH3p* promoter
THD3p-R	CAACTTGTAGTTCATCATGTTCTTCAT TTTGTTTGTTTATGTGTGTTTATTCGA AAC	Amplification of *TDH3p* promoter
AtATR2-F	TTACCAAACATCTCTCAAGTATCTAC CG	Amplification of *AtATR2* fragment
AtATR2-R	ATGAAGAACATGATGAACTACAAGT TG	Amplification of *AtATR2* fragment
SDH1t-F	GGTAGATACTTGAGAGATGTTTGGTA ATTTGAACCTCATTGTATTTTACGG	Amplification of *SDH1t* terminator
SDH1t-R	GATTCATACAATTTGCACTATTCCGG CGACAGCACCCTTGTACAGCAATTC	Amplification of *SDH1t* terminator
SED1p-F	TCTACCTTCCATACACCACTGATTG	Amplification of *SED1p*

		promoter
SED1p-R	CATCTTAATAGAGCGAACGTATTTTA TTTTG	Amplification of *SED1p* promoter
CYB5-F	AATAAAATACGTTCGCTCTATTAAGA TGATGCCTAAAGTTTACAGTTACCAA G	Amplification of *CYB5* fragment
CYB5-R	CTCTCCTTCCTATTGGATTGAAGTTTT ATTCGTTCAACAAATAATAAGCAAC AC	Amplification of *CYB5* fragment
PDB1t-F	AACTTCAATCCAATAGGAAGGAGAG	Amplification of *PDB1t* terminator
PDB1t-R	ATGAAGCAGCCAATACCTGTTTTACA ACTTTCGCTTAGGTTCCATGCC	Amplification of *PDB1t* terminator
FjTAL-F	ATGAACACCATTAATGAATACTTGAG	Amplification of *FjTAL* fragment
FjTAL-R	TTAATTGTTAATCAAATGATCCTTAA CC	Amplification of *FjTAL* fragment
GAL7p-F	TTTGCCAGCTTACTATCCTTCTTG	Amplification of *GAL7p* promoter
GAL7p-R	CTCAAGTATTCATTAATGGTGTTCATT TTTGAGGGAATATTCAACTGTTTTTTT	Amplification of *GAL7p* promoter

DIT1t-F	TTAAGGATCATTTGATTAACAATTAA ATAAAGTAAGAGCGCTACATTGG	Amplification of *DIT1t* terminator
DIT1t-R	ACTTGTTACTCCGCAACGC	Amplification of *DIT1t* terminator
	**Construction of the synthesis pathway for naringenin**	
GAL10p-2-F	*TTATATTGAATTTTCAAAAATTCTTACT TTTTTTTTGGATGGACGR*	Amplification of *GAL10p* promoter
GAL10p-2-R	*CATTGTACTTCAATCTATAGTTTTTTCT CCTTGACGTTAAAGTATAGAG*	Amplification of *GAL10p* promoter
PhCHS-F	*CAAACCTCTGGCGAAGAATTGTTAATT AAAGATCTCCGCGGTCCG*	Amplification of *PhCHS* fragment
PhCHS-R	*GAATTTTTGAAAATTCAATATAAATGG TTACGGTGGAAGAATACCGC*	Amplification of *PhCHS* fragment
ALD5p-F	*CTATAGATTGAAGTACAATGACGCTAA CACTAAGTTATG*	Amplification of *ALD5p* promoter
ALD5p-R	*TTCTTCTTCTTTGGCTTTTTGTTAATGTT GTG*	Amplification of *ALD6p* promoter
MsCHI-F	*CAAAAAGCCAAAGAAGAAGAAATGGC AGCAAGCATTACGGC*	Amplification of *MsCHI* fragment
MsCHI-R	*GACTGAATCAAATGTAATCCACCTAGG TCAGTTACCGATTTTAAAGGC*	Amplification of *MsCHI* fragment

ARO7p-F	*TGGATTACATTTGATTCAGTCATACACGAATTATG*	Amplification of *ARO7p* promoter
ARO7p-R	*ATCTTATACCAATTTTATGCAGGATGCTGAGTG*	Amplification of *ARO7p* promoter
Pc4CL-F	*GCATAAAATTGGTATAAGATATGGGTGACTGCGTTGCCCC*	Amplification of *Pc4CL* fragment
Pc4CL-R	*TCGACGGTATCGATAAGCTTTTACTTCGGCAGGTCGCCGC*	Amplification of *Pc4CL* fragment
LEU2-F	TTCGCCCTATAGTGAGTCGTATTACAACTGTGGGAATACTCAGGTATCGTAAG	Amplification of *LUE2* fragment
LEU2-R	TTAAGCAAGGATTTTCTTAACTTCTTCGGC	Amplification of *LUE2* fragment
	**Construction of the synthesis pathway for genistein**	
RPY26-F	TCTAGAACTAGTGGATCCCCCGGG	Amplification of pY26 vector
RPY26-R	GAGCTCCAGCTTTTGTTCCCTTTAGT	Amplification of pY26 vector
SED1p-F	CTAAAGGGAACAAAAGCTGGAGCTCAATTATCTCCCAGACGGCACCG	Amplification of *SED1p* promoter
SED1p-R	CTTAATAGAGCGAACGTATTTTATTTTGCTTGTC	Amplification of *SED1p* promoter

Ter22-F	AATGTCAAAAGCCTCAAGGTGCC	Amplification of *Ter22* terminator
Ter22-R	ATAGGTTGGCTTCCATGTTGGCTAT	Amplification of *Ter22* terminator
TDH1p-F	TTTGTTTTGTGTGTAAATTTAGTGAAG TACTG	Amplification of *TDH1p* promoter
TDH1p-R	GCACCGGCCTCATCGTCTTCATGTCC CACCAGCCAACACTTG	Amplification of *TDH1p* promoter
INO1p-F	GAAGACGATGAGGCCGGTGC	Amplification of *INO1p* promoter
INO1p(Gm)-R	CAATTCCTTAACGATTTCCTTAGCCA TTGTTACTTCTTTTTCACTGGAAAAAA AAGGG	Amplification of *INO1p* promoter
INO2p(Ge)-R	GTGGTAGTAGAAGTAGAAGAAGCCA TTGTTACTTCTTTTTCACTGGAAAAAA AAGGG	Amplification of *INO1p* promoter
INO1p(Pl)-R	TTCCTTATTAGAGTTTTCGTTAGCCAT TGTTACTTCTTTTTCACTGGAAAAAA AAGGG	Amplification of *INO1p* promoter
GeHIS-F	CAAAATAAAATACGTTCGCTCTATTA AGATGTTGGTTGAATTGGCTATCACT TTGTTAG	Amplification of *GeHIS* fragment

GeHIS-R	GCACCTTGAGGCTTTTGACATTTTATGAAGAAAACAATTTTGGAACTGCTGAAGA	Amplification of *GeHIS* fragment
GmIFS-F	GCAAAATAAAATACGTTCGCTCTATTAAGATGTTGTTAGAATTAGCTTTGGGTTTATTC	Amplification of *GmIFS* fragment
GmIFS-R	GGCACCTTGAGGCTTTTGACATTTTAGGACAACAATTTAGAAGCAACACCAAT	Amplification of *GmIFS* fragment
GmIFS2-F	GCAAAATAAAATACGTTCGCTCTATTAAGATGTTGTTGGAATTGGCTTTGGGT	Amplification of *Gm2IFS* fragment
GmIFS2-R	GCACCTTGAGGCTTTTGACATTTTAAGACAATAACTTAGAAGCAACACCAATTCTAGC	Amplification of *Gm2IFS* fragment
GuIFS-F	CAAAATAAAATACGTTCGCTCTATTAAGATGTTGGTTGAATTGGCTATCACCTTG	Amplification of *GuIFS* fragment
GuIFS-R	GCACCTTGAGGCTTTTGACATTTTATGAAGAGAACAACTTTGGGACAGC	Amplification of *GuIFS* fragment

MtIFS-F	GCAAAATAAAATACGTTCGCTCTATTAAGATGTTGGTTGAATTAGCCGTTACTTTGTT	Amplification of *MtIFS* fragment
MtIFS-R	GCACCTTGAGGCTTTTGACATTTTAGGAAGATAACAACTTATCAGCAACACC	Amplification of *MtIFS* fragment
PlIFS-F	CAAAATAAAATACGTTCGCTCTATTAAGATGTTATTAGAATTAGCTTTAGGTTTGTTAG	Amplification of *PlIFS* fragment
PlIFS-R	GCACCTTGAGGCTTTTGACATTTTAAGACAAAACCTTAGCAGCGACG	Amplification of *PlIFS* fragment
TpIFS-F	GCAAAATAAAATACGTTCGCTCTATTAAGATGTTGTTGGAATTGGCCTTGGG	Amplification of *TpIFS* fragment
TpIFS-R	GCACCTTGAGGCTTTTGACATTTTAAGACAACAACTTAGAAGCAACACCAATTC	Amplification of *TpIFS* fragment
GmCPR-F	ATAGCCAACATGGAAGCCAACCTATTTACCAAACATCTCTCAAGTATCTACCAGTAG	Amplification of *GmCPR* fragment
GmCPR-R	CAGTACTTCACTAAATTTACACACAAAACAAAATGCAAGATTCTGGTTCCAT	Amplification of *GmCPR* fragment

	GAAGATTTC	
AtCPR-F	ATAGCCAACATGGAAGCCAACCTAT TTACCAAACGTCACGCAGGTAAC	Amplification of *AtCPR* fragment
AtCPR-R	CAGTACTTCACTAAATTTACACACAA AACAAAATGAGCTCTAGCTCTTCTTC TTCTACC	Amplification of *AtCPR* fragment
CrCPR-F	ATAGCCAACATGGAAGCCAACCTAT TTACCAAACATCTCTTAAGTATCTAC CTGTC	Amplification of *CrCPR* fragment
CrCPR-R	GTACTTCACTAAATTTACACACAAAA CAAAATGGATTCTTCATCTGAAAAGT TGTCACC	Amplification of *CrCPR* fragment
LjCPR-F	ATAGCCAACATGGAAGCCAACCTAT TTACCAGACATCCCTCAAGTACCTAC C	Amplification of *LjCPR* fragment
LjCPR-R	GTACTTCACTAAATTTACACACAAAA CAAAATGGAGGAATCCTCTTCTATGA AGATCAG	Amplification of *LjCPR* fragment
GmHID-F	ATGGCTAAGGAAATCGTTAAGGAAT TGTTG	Amplification of *GmHID* fragment

GmHID-R	CCGGGGGATCCACTAGTTCTAGATTAAACCAAAAATGAAGCTAATCTCTTGATCATTG	Amplification of *GmHID* fragment
GeHID-F	ATGGCTTCTTCTACTTCTACTACCACTTC	Amplification of *GeHID* fragment
GeHID-R	CCGGGGGATCCACTAGTTCTAGATTAAACCAAGAAAGAAGCAATTCTACCAATCAAA	Amplification of *GeHID* fragment
PlHID-F	ATGGCTAACGAAAACTCTAATAAGGAAATTGTTAAG	Amplification of *PlHID* fragment
PlHID-R	CGGGGGATCCACTAGTTCTAGATTAAACCAAGAAGGAAGCTAATCTCTTGATCAT	Amplification of *PlHID* fragment
	**Modification of the genistein pathway**	
RPRS424-F	CAGCTTTTGTTCCCTTTAGTGAGGG	Amplification of pRS424 vector
RPRS424-R	CGCGCGCTCACTGGCCGTCG	Amplification of pRS424 vector
SED1p-(PRS424)-F	CGACGGCCAGTGAGCGCGCGAATTATCTCCCAGACGGCACCG	Amplification of *SED1p* promoter
SED1p-R	CTTAATAGAGCGAACGTATTTTATTTTGCTTGTC	Amplification of *SED1p* promoter

Ter22-F	AATGTCAAAAGCCTCAAGGTGCC	Amplification of *Ter22* terminator
Ter22-(PRS424)-R	CCCTCACTAAAGGGAACAAAAGCTGATAGGTTGGCTTCCATGTTGGCTAT	Amplification of *Ter22* terminator
STB5-F	GACAAGCAAAATAAAATACGTTCGCTCTATTAAGATGGATGGTCCCAATTTTGCACATC	Amplification of *STB5* fragment
STB5-R	GGCACCTTGAGGCTTTTGACATTTCATACAAGTTTATCAACCCAAGAGACGTC	Amplification of *STB5* fragment
YEF1-F	GCAAAATAAAATACGTTCGCTCTATTAAGATGAAAACTGATAGATTACTGATTAACGCT	Amplification of *YEF1* fragment
YEF1-R	GGCACCTTGAGGCTTTTGACATTTTAGATTGCAAAATGAGCCTGACGAG	Amplification of *YEF1* fragment
EcPntAB-F	GACAAGCAAAATAAAATACGTTCGCTCTATTAAGTGGAAGGGAATATCATGCGAATTGG	Amplification of *EcPntAB* fragment
EcPntAB-R	GGCACCTTGAGGCTTTTGACATTTTACAGAGCTTTCAGGATTGCATCCAC	Amplification of *EcPntAB* fragment

ALD6-F	CAAGCAAAATAAAATACGTTCGCTC TATTAAGATGACTAAGCTACACTTTG ACACTGCT	Amplification of *ALD6* fragment
ALD6-R	GGCACCTTGAGGCTTTTGACATTTTA CAACTTAATTCTGACAGCTTTTACTTC AGTG	Amplification of *ALD6* fragment
HEM2-F	GCAAAATAAAATACGTTCGCTCTATT AAGATGCATACAGCTGAATTTTTGGA AACAG	Amplification of *HEM2* fragment
HEM2-R	GGCACCTTGAGGCTTTTGACATTTTA GTTTTCTTCATCTAACCAGTCTAGGA ACTC	Amplification of *HEM2* fragment
HEM3-F	GCCAACATGGAAGCCAACCTATTCA TTTGATTCTGTCTAAATTAATTTCATC CAG	Amplification of *HEM3* fragment
HEM3-R	TTAGTTTCGAATAAACACACATAAAC AAACAAAATGGGCCCTGAAACTCTA CATATTGG	Amplification of *HEM3* fragment
TDH3p-F	TTTGTTTGTTTATGTGTGTTTATTCGA AACTAAGTTC	Amplification of *TDH3p* promoter
TDH3p-R	CCCTCACTAAAGGGAACAAAAGCTG TCATTATCAATACTGCCATTTCAAAG	Amplification of *TDH3p* promoter

	AATACG	
ACC1p-F	CAATAATCAGAGATTACAGTCGGCA TCAATTTTCCTTTCCTTATTCTACTCTT TTTATC	Amplification of *ACC1p* promoter
ACC1p-R	GGTCTTGTGGAGTAAGCGTCCATGGT AGAAACTTGATTTTTTCTAATTTTCTG CGC	Amplification of *ACC1p* promoter
ZWF1p-F	CAATAATCAGAGATTACAGTCGGCA TCAAGTACATAGTGACATTTAAATAA TAGCAAG	Amplification of *ZWF1p* promoter
ZWF1p-R	GGTCTTGTGGAGTAAGCGTCCATCTT GCCTTATGTGGTTTTCTATTCTATTGG ATTTAC	Amplification of *ZWF1p* promoter
RPL3p-F	CGACAATAATCAGAGATTACAGTCG GCATGCGAACAAACCAGCTGCTAAG	Amplification of *RPL3p* promoter
RPL3p-R	GGTCTTGTGGAGTAAGCGTCCATGAT TGATTGTTGTAGTAACTGTGTTGTTCT AGAG	Amplification of *RPL3p* promoter

TEF1p-F	CAATAATCAGAGATTACAGTCGGCAGGTGTCGTTAATTACCCGTACTAAAGG	Amplification of *TEF1p* promoter
TEF1p-R	GTCTTGTGGAGTAAGCGTCCATTTTGTAATTAAAACTTAGATTAGATTGCTATGCTTTC	Amplification of *TEF1p* promoter
ITR1p-F	CAATAATCAGAGATTACAGTCGGCATAACATAATAAGCGCATATACTTAGTTCTCTCC	Amplification of *ITR1p* promoter
ITR1p-R	GGTCTTGTGGAGTAAGCGTCCATTTTAACACCCACTGCAGAAACAAAGAAAATG	Amplification of *ITR1p* promoter
CCW12p-F	CAATAATCAGAGATTACAGTCGGCAAACGCCACCAAAAAAAAAAAAAATAAAAGCCAATC	Amplification of *CCW12p* promoter
CCW12p-R	GGTCTTGTGGAGTAAGCGTCCATTATTGATATAGTGTTTAAGCGAATGACAGAAG	Amplification of *CCW12p* promoter
	**Glycosylation of genistein**	
GmUGT4-F	GCAAAATAAAATACGTTCGCTCTATTAAGATGACAATGAAGGATTCTATCGTTTTGTAC	Amplification of *GmUGT4* fragment

GmUGT4-R	GACCAATGTAGCGCTCTTACTTTATTTAATGTTCTCTCCACAATTCAACCAACTTATTC	Amplification of *GmUGT4* fragment
PlUGT43-F	GCAAAATAAAATACGTTCGCTCTATTAAGATGACTAGATACGAAGTTGTTTTTATTGC	Amplification of *PlUGT43* fragment
PlUGT43-R	AATGTAGCGCTCTTACTTTATTTAAGATGTTAATTCTTGAATTAATGAAACCAAATTAG	Amplification of *PlUGT43* fragment
DIT1t(PRS424)-F	CGACGGCCAGTGAGCGCGACTTGTTACTCCGCAACGCTTTTC	Amplification of *GmUGT4/PlUGT43* fragment
SED1p(ADH1t)-R	CCAATTCAGCTGGCGTAATAGCAATTATCTCCCAGACGGCACCG	Amplification of *GmUGT4/PlUGT43* fragment
SED1p(CYC1t)-R	CCAATTCGCCCTATAGTGAGTCGTATTACAATTATCTCCCAGACGGCACCG	Amplification of *GmUGT4/PlUGT43* fragment
DIT1t-F	ATAAAGTAAGAGCGCTACATTGGTCTACC	Amplification of *DIT1t* terminator
DIT1t-R	CACGACGTTGTAAAACGACGGCACTTGTTACTCCGCAACGCTTTTC	Amplification of *DIT1t* terminator

Homology arms of integration sites

XI-5-up-F	GCGGAGAAGTCGTTGATAGC	Amplification of *XI-5-up* fragment
XI-5-up-R	TGGTGCACGGAGTTTATGG	Amplification of *XI-5-up* fragment
XI-5-down-F	TTGGATTACATCCTACTTTT	Amplification of *XI-5-down* fragment
XI-5-down-R	GCCGGAATAGTGCAAATTGT	Amplification of *XI-5-down* fragment
XI-2-up-F	TAACTCTTCGTATGAGGATTTTCGATG	Amplification of *XI-2-up* fragment
XI-2-up-R	AAGGATAGTAAGCTGGCAAATTCTATGGCACATTTTTCTGTTGAG	Amplification of *XI-2-up* fragment
XI-2-down-F	CGTTGCGGAGTAACAAGTCCACAAGTAAAGCTCGTTGAC	Amplification of *XI-2-down* fragment
XI-2-down-R	ATGGTTGAAAAGGTTACAGAGGATC	Amplification of *XI-2-down* fragment
Ty3-up-F	TGTTGTATCTCAAAATGAGATATGTCAGTATGACAATACG	Amplification of *Ty3-up* fragment
Ty3-up-R	GCTCCAATTCAGCTGGCGTAATAGCTCGGATCTAAACTAATTGTTCAGGCATTTATACT	Amplification of *Ty3-up* fragment

Ty3-down-F	AGAAGTTAAGAAAATCCTTGCTTAA GATTCCGCGCTTCCACCACT	Amplification of *Ty3-down* fragment
Ty3-down-R	TGTTGTATTACGGGCTCGAGTAATAC C	Amplification of *Ty3-down* fragment
EXG1-up-F	ACGAGCCTGAGACAAGCCCG	Amplification of *EXG1-up* fragment
EXG1-up-R	TTTAGTTGGTAATTAACTAGAAAAAG AAAGTAAACAAAAAATCAAAGG	Amplification of *EXG1-up* fragment
EXG1-down-F	GTCGCCCTCAGTCCGCTCATTTTAG	Amplification of *EXG1-down* fragment
EXG1-down-R	GTTGTTTAAGTTCTTTTATCCTTCCTT AGATAACACC	Amplification of *EXG1-down* fragment
Ty1-up-F	TGTTGGAATAAAAATCAACTATCATC TACTAACTAGTATTTACGTTAC	Amplification of *Ty1-up* fragment
Ty1-up-R	TATGTTAATATTCATTGATCCTATTAC ATTATCAATCCTTGCG	Amplification of *Ty1-up* fragment
Ty1-down-F	CTTTTTTTTTCTCTTGAACTCGATAAA ACGGAATGATGAATAATATTTATAG AATTGTG	Amplification of *Ty1-down* fragment
Ty1-down-R	TGAGAAATATGTGAATTTTGAGATAA TTGTTGGGATTC	Amplification of *Ty1-down* fragment

Ty2-up-F	TGTTGGAATAAAAATCAACTATCATC TACTAACTAGTATTTACGTTAC	Amplification of *Ty2-up* fragment
Ty2-up-R	GTTAATATTCATTGATCCTATTACATT ATCAATCCTTGCG	Amplification of *Ty2-up* fragment
Ty2-down-F	CGAATATAAAATGATGATAATAATA TTTATAGAATTGTGTAGAATTGCAG	Amplification of *Ty2-down* fragment
Ty2-down-R	GAGAATGTGGATTTTGATGTAATTGT TGGG	Amplification of *Ty2-down* fragment
FAS1p-up-F	TACTATGCGGTCTCGTCCTCTACG	Amplification of *FAS1p-up* fragment
FAS1p-up-R	TGCCGACTGTAATCTCTGATTATTGTC G	Amplification of *FAS1p-up* fragment
FAS1p-down-F	ATGGACGCTTACTCCACAAGACC	Amplification of *FAS1p-down* fragment
FAS1p-down-R	TCGGTGTTACCTTGACCACCG	Amplification of *FAS1p-down* fragment
OCA5-up-F	GGCGCAATATGCAGGTACCG	Amplification of *OCA5-up* fragment
OCA5-up-R	GTAGTATATAATTCTAAAGGAAATA ATTTCAACTTTATTTGCTAGCGAGCC CTTTACC	Amplification of *OCA5-up* fragment

OCA5-down-F	AAAGTTGAAATTATTTCCTTTAGAAT TATATACTACTTCTAC	Amplification of *OCA5-down* fragment
OCA5-down-R	TATCTCGATAGTTGGAAGATTTAGTA CCATAAGTAGC	Amplification of *OCA5-down* fragment

Table S4 Codon optimized exogenous gene sequences

Synthetic gene	Nucleotide Sequence (5′ - 3′)
AtC4H	TTAACAGTTTCTTGGCTTCATAACAATAATAGAGTGGTTCAAA<br>ATGTGCAAAGAGAATTGACCACCCTTTTCAGAGGTGTCGACCT<br>TAGATTGACCTGGTGGTGGCAACAATTCAAAATTTTGGACCAT<br>TCTACCAATGGTAATACCTAAAATTGGCAAAGCCAAAATAAT<br>ACCAGGACAAGATCTTCTACCAACACCAAATGGGACGTATCT<br>AAAATCATTACCATTAGCTTCAACGTGAGATTCTTCTTCGAAA<br>AATCTTTCTGGTCTGAATTCTTCTGGTTTCTTCCATGAATTTGGG<br>TTGTTAGCCAACCACCAAGCGTTAACCAAAATCTTACTTTCAG<br>CAGGGATATCGTAACCAGCTAACTTAGCATCATGCAAATTCAT<br>ATGTGGGACTAACAAAGGAATAGCCATTCTCAAACGCAAAGT<br>TTCCTTAACAACTGCTTGCAAATATGGCAATTTATGCAAGTCA<br>GGTTCAGTAACTTGGACACCAGGACCTAAAACAGTATCTAATT<br>CATTTCTCAACTTAGATTGAATTTCAGGATGATTAACTAATTCA<br>GCAATACCCCATTCAATGGACCATAAAGTAGTTTCAATGGCAG<br>CAACGTTAATATTCTCAACAATATACAAAACATTATCTTCATTA<br>ATTTCACCCTTTTGCTCGGCTTCCAAAATGTGGTCGATAGCACA<br>CTTAAGACCTTCAGAACCAGTTGGCTTTGAAGAGGCAATTTGC<br>TTACGTTCATCAACAAAGTACTTCTTAAACAAAGCAATTCTTCT<br>ATCCTTAACATCTTGACAAATCTTTAGGTAACCTCTTAAAAATG<br>GTCTTAAAATTGGAATGAAGTCACCGTAGTTGTATTCGAATGA<br>TTGGGCCAATCTGGATCTTTCACCGTTCAAAGCCTTCAAACGC<br>AAGAATAAAGGATCATCTTCTGATTCAAATCTACGGTCGAACA<br>TAATTCTAAACATGTTATTGTACATCATCAATTGTAATCTCTTTC<br>TCAAAACGATACCCTTGGTAGCAGAATCAGGATTCTTCTTAAC<br>ATCTTCGACAACGGAAGCAGCTTCAAATTCCCAACCTTCTCTA<br>TTTTGTTGAACAACTTTGTTAGTGAAGAAAGGGACGGTCATTAT<br>TCTTCTCATCTTACGCCAGTGTTCACCGTAAACTGTGAAAACCA<br>TGTCTTGACCTTTACCAGTGAAAATATCAAAAACAACGTTTCT<br>AGTTCTAGAACCGAATTCAACACCTTGAGTTAACAAAACCTCC<br>TTAGTCAAATCTGGAGAAGAAACAACAACCAAATTTCTTTGGC<br>CCATTCTCAATAAGAACAAGTCACCGAACTTTTTAGCGTAATC<br>AACCAAGTTTCTATGGTTCAAATCGTCACCAACTTGTAACCAG<br>TTACCGAAAATTGGAATTGGGATTGGACCTGGTGGCAACTTCA<br>ATTTTTTACCTCTCAACTTGGAAATGACAGTGGCCAAAATAAC<br>AGCGACGAAAACGGCAATCAAAGACTTTTCCAACAACAACAA<br>ATCCAT
AtPAL2	ATGGATCAAATTGAAGCCATGTTGTGTGGTGGTGGTGAAAAAA<br>CTAAAGTCGCAGTCACTACCAAGACCTTGGCTGATCCTTTGAA<br>CTGGGGTTTAGCTGCTGATCAAATGAAGGGTTCTCATTTGGATG

AAGTTAAGAAGATGGTTGAAGAATATCGTAGACCAGTTGTTAA
CCTAGGTGGCGAAACATTGACCATTGGTCAAGTTGCTGCTATC
TCTACTGTTGGTGGTTCCGTTAAAGTTGAATTAGCTGAAACTTC
AAGAGCTGGTGTTAAAGCTTCTTCTGACTGGGTTATGGAATCT
ATGAACAAAGGTACTGATTCTTATGGTGTTACTACTGGTTTTGG
TGCTACTAGTCATAGAAGAACTAAGAATGGTACAGCTTTGCAA
ACTGAATTGATCAGATTTTTGAACGCTGGTATTTTCGGTAACAC
TAAGGAAACTTGTCATACCCTACCACAATCTGCCACTCGTGCT
GCTATGTTGGTTAGAGTAAACACCTTATTGCAAGGTTATTCTGG
TATTAGATTCGAAATCTTGGAAGCCATTACATCTTTGTTAAATC
ATAACATCTCTCCTTCCTTGCCATTGAGAGGTACTATTACTGCT
TCTGGTGACTTAGTTCCATTGTCATACATTGCCGGTTTGTTGAC
CGGTAGACCAAACTCTAAGGCTACTGGTCCAGATGGTGAATCA
TTGACCGCTAAGGAAGCTTTCGAAAAGGCTGGTATTAGTACTG
GTTTCTTTGACTTGCAACCAAAAGAAGGGTTAGCTTTAGTCAA
CGGTACTGCTGTCGGTTCCGGTATGGCCTCAATGGTTTTGTTTG
AAGCTAACGTTCAAGCCGTTTTGGCTGAAGTCTTATCTGCTATT
TTCGCTGAAGTTATGTCCGGCAAACCAGAATTCACTGATCATTT
GACTCATAGATTGAAACACCATCCAGGTCAGATTGAAGCTGCT
GCTATTATGGAACATATTTTGGATGGTTCTTCTTACATGAAGCT
AGCTCAAAAGGTACATGAAATGGATCCATTACAAAAGCCAAA
GCAAGATAGATACGCACTTAGAACTTCTCCACAATGGTTGGGT
CCACAAATTGAAGTTATTAGACAAGCTACTAAATCTATTGAAA
GAGAAATTAACTCTGTCAATGATAATCCATTGATTGATGTTTCC
AGAAACAAAGCTATTCACGGTGGTAATTTCCAAGGTACTCCAA
TTGGTGTTTCTATGGATAATACTAGATTAGCTATAGCTGCCATT
GGTAAATTAATGTTTGCCCAATTTTCTGAATTGGTTAATGATTT
CTACAACAATGGTTTGCCATCAAACTTAACAGCTTCTTCTAACC
CATCTTTGGATTACGGTTTTAAGGGTGCTGAAATTGCCATGGCT
TCTTACTGTTCTGAATTACAATACTTGGCTAACCCAGTTACCTC
CCATGTTCAATCAGCTGAACAACACAACCAAGATGTTAACTCT
TTGGGCCTAATTTCCTCTAGAAAGACTTCTGAAGCTGTTGATAT
CTTAAAGTTAATGTCTACAACTTTCTTAGTTGGTATCTGTCAAG
CTGTTGACTTGAGACATTTGGAAGAAAATTTGAGACAAACAGT
TAAGAATACTGTTAGTCAAGTTGCTAAAAAAGTTTTGACTACT
GGTATTAACGGTGAATTGCATCCTAGTAGATTCTGTGAAAAGG
ATTTGTTAAAAGTCGTTGATAGAGAACAAGTTTTCACATACGTT
GATGATCCATGTTCAGCTACCTACCCTTTAATGCAAAGATTAA
GACAAGTTATTGTTGATCATGCCTTATCTAATGGTGAAACTGA
AAAGAACGCTGTCACTTCTATTTTTCAAAAGATTGGTGCCTTTG
AAGAAGAATTGAAGGCTGTTTTGCCAAAGGAAGTTGAAGCAG
CTAGAGCTGCATATGGTAATGGTACCGCTCCTATTCCTAATAG
AATTAAGGAATGTAGAAGTTATCCATTGTATAGATTCGTTAGA
GAAGAACTAGGTACTAAATTGTTGACTGGTGAAAAAGTCGTTT

	CTCCAGGTGAAGAATTTGATAAGGTTTTTACAGCTATGTGTGA<br>AGGAAAGTTAATTGATCCATTAATGGATTGCTTAAAGGAATGG<br>AACGGTGCTCCAATTCCAATTTGTTAAAT
AtATR2	TTACCAAACATCTCTCAAGTATCTACCGGAGGTTTGCAAGTTCT<br>TAACGAAACCTTCAGCTTTAGTTGAGTCCATAGAACCTTGTTCT<br>TGAGCAATAGTGTGCAAAGATCTATGAACATCTCTAGCCATAC<br>CTTTAGCATCGCCACAAACATACAAGTAAGCACCTTGAGAAAT<br>CATGTTCCAGATGTCAGAAGCTTTATCCATCATCTTATGTTGAA<br>CGTATTCCTTAGTTGGACCTTCTCTTGAGAAAGCAACTGACAAT<br>TCAGCTAAAGCACCAGATTCAACAAATCTTTGCAATTCTTCTTC<br>ATAGATAAAATCCATTCTTCTATTTCTGCAACCGAAGAACAAA<br>ACAGATGGACCAAGTTCAACACCAGATTCAACTAAAGCCAAT<br>CTTTCTTGCAAGAAACCTCTGAATGGTGCTAAACCAGTACCTG<br>GACCAATCATGATGATTGGGACCTTAGAATCAGATGGTAACTT<br>AAAATTAGATTGTCTAACAAAAATTGGAGCAGAAGAACAATT<br>TTCAGACTTTTCATATGGAACAGCATTCTTCATCCAAGTAGAAC<br>AGACACCCTTATGGATTCTACCGGTTGGCATTTTTTCATAGACT<br>AAAGCGCAGGTAACATGAATTCTTGTTTCAGCGATTTTTGGGG<br>AGCTAGAGATAGAATAGAATCTTGGCTGTAATCTTGGGGCGAC<br>ACCGGCAAAGAACACACCCAATGGTGGTTTAGCAGATGGGAA<br>TTCAGCCATAACTTCTAGTAATGATCTTTGAGATTCAACAACCC<br>ACTTGGAGTATTCATCCTTTCCAGCTGGGGAAGCCAAATGTTTC<br>AATCTTTCGGCTTCAGTTGGATCACTAGCGTGTGCGGCTAGGG<br>CAACTAGGGCTGACTTTTTAGGAGAGGATAGCAAACAAGCGT<br>ATCTAGTTAAAGCGGTTCTAAGATTACATGGAGGGAATGGAGG<br>TGGCAAAGAAGAGGAGATCGGAGTACCATCTTCCTTTTCAGCA<br>TGCAAAGAAAAATAGGTGTCAGGAGACATATCCAATAATCTC<br>AAGGCTTCATCAACAGTTTCGGACAAGTTGTCACACAAAACAC<br>CAACATGGTCACCAGTTTCGTAAGTTAAACCAGAACCGGCTAT<br>ATCGAATTCTAGGTGGATACAAGAACGATCAGATTCTGGAGTA<br>TGTAATTCTCTTTTGACTGCTACATTAGCCTTATAAGGATGTTG<br>AGCATCAAAAACGGTGTAACCATTGCCGTTAGCCATGTTTATA<br>TCGTTAAACTTAGCATCTTCAGAATCATGGATACTAACTCTATA<br>TTCTAAGACAGCTGCAGTATATGGTGTAGCAACAGCGGTGTCA<br>CCTTCTTCTCTTAGAATGGTATCTAGTTCTGGCCACAAGGCTTC<br>ACGCCATGCGGTGAAGTCATCTTCAATACATTGATCATCATCC<br>CCCAAACCAACTTGAACCAATCTTTGAGCACCTTGTTCGACCA<br>AAATGTCATCAACAACCTTAGCGACCTTATTAAAATGTTCGTA<br>TTGTCTATTACCCAAACCAAAAACCCCATATTTTAAGTTCTTCA<br>ACCATTCACCTCTATCGTTACCTTCGGTGAACCACTTATAAAAT<br>CTGGCAGCGTTATCAGTAGGTTCACCGTCACCGTAAGTAGCTA<br>AAAAGAAAAAGGCAACATCTTCTTTCTTTAGCTTTTCCTCATAT<br>TCATCGTCGTCAGCGGCGTAATCATCCAAATCAACGATCTTGA<br>ATCTAGTCTTTTCATATCTAGCCTTAGCTTCTTCACCCAAAGCC

	TTAGCGAAACCTTCAGCAGTACCAGTTTGAGTACCGAAGAAA<br>ATAGTAACTTTCTTTCTACCGTCATCAATTTCTTCTTCTCTTGGC<br>TTTATAACCAAAGGTTTTAAAGGTTCAACTCTCTTAGAGTTACC<br>CGAACCTGAACGCCTCCAAACTAGCATAACAATACAACCAAT<br>CAAAACAGCGATGGAAGTAGTGACAATCATAGCAAATTGTCT<br>ATTTTCAATCAACATAGAACTAAGTTCAGCGGCGACAGATTCG<br>TATGCAGAAGCATTAGCGGGATCGGAAACAATAACTGGTTCA<br>CCCTTTATAATGGCAGCCATCAAGTCGATCATGGAAGTAGAGG<br>AGGAAGAACTAGAGGACATTGCATTGTAGATGTTATAAGAAG<br>AAGAAGATAATAATAAGAAAAAGAAAGAAGAAGATGGCAAC<br>AACAAGTCTCTTTCAGTATGAATAGTTGGTGGCTTAGTCAAGT<br>GTGGAGTTGGAGACAAGGAAACACCCTTACTATTCTTAGAAAC<br>ACTACACAATTTCAACTTGTAGTTCATCATGTTCTTCAT
FjTAL	ATGAACACCATTAATGAATACTTGAGTTTAGAAGAATTCGAAG<br>CAATAATCTTCGGTAACCAAAAAGTAACTATCTCTGATGTTGT<br>CGTAAACAGAGTTAACGAAAGTTTTAACTTCTTAAAGGAATTT<br>TCTGGTAATAAGGTTATATATGGTGTAAACACTGGTTTCGGTCC<br>AATGGCTCAATACAGAATCAAGGAATCTGATCAAATCCAATTG<br>CAATACAATTTGATAAGAAGTCATTCTTCAGGTACTGGTAAAC<br>CATTATCTCCTGTTTGTGCTAAGGCTGCAATCTTGGCAAGATTG<br>AACACATTGTCTTTAGGCAACTCAGGTGTTCACCCATCTGTTAT<br>TAATTTGATGTCTGAATTGATAAACAAAGACATCACTCCTTTG<br>ATATTCGAACATGGTGGTGTTGGTGCATCTGGTGACTTGGTCCA<br>ATTGTCCCACTTGGCCTTAGTATTGATAGGTGAAGGTGAAGTTT<br>TCTATAAAGGTGAAAGAAGACCAACACCTGAAGTCTTCGAAA<br>TCGAAGGTTTAAAGCCTATACAAGTAGAAATCAGAGAAGGTTT<br>AGCTTTGATTAATGGTACTTCTGTCATGACAGGTATAGGTGTTG<br>TCAACGTATACCATGCTAAGAAATTGTTGGATTGGTCATTGAA<br>GTCCAGTTGTGCCATTAATGAATTGGTTCAAGCATATGATGAC<br>CATTTCTCTGCAGAATTGAACCAAACCAAGAGACACAAGGGT<br>CAACAAGAAATCGCATTGAAGATGAGACAAAATTTGTCCGAT<br>AGTACATTGATCAGAAAGAGAGAAGACCACTTATACTCAGGT<br>GAAAACACCGAAGAAATTTTCAAAGAAAAGGTTCAAGAATAC<br>TACTCCTTGAGATGCGTCCCACAAATCTTGGGTCCTGTATTGGA<br>AACTATTAATAACGTTGCCTCAATCTTGGAAGATGAATTCAAT<br>TCCGCTAACGATAACCCAATCATCGACGTTAAAAATCAACATG<br>TTTATCACGGTGGTAACTTCCATGGTGACTACATTTCTTTAGAA<br>ATGGACAAATTGAAGATAGTTATCACAAAATTGACCATGTTGG<br>CTGAAAGACAATTGAACTACTTGTTGAACTCAAAGATTAACGA<br>ATTGTTGCCACCTTTCGTTAATTTGGGTACATTGGGTTTTAACTT<br>CGGTATGCAAGGTGTTCAATTCACCGCCACTTCAACTACAGCT<br>GAATCCCAAATGTTGAGTAACCCAATGTACGTTCATTCCATCC<br>CTAACAACAACGATAACCAAGACATCGTCTCTATGGGTACCA<br>ACTCAGCCGTCATTACTTCCAAAGTAATAGAAAACGCATTCGA

	AGTTTTGGCCATCGAAATGATCACAATTGTCCAAGCTATCGAT TACTTGGGTCAAAAGGACAAGATCTCTTCTGTTTCTAAGAAAT GGTACGATGAAATAAGAAACATAATCCCAACCTTTAAGGAAG ACCAAGTTATGTACCCTTTCGTACAAAAGGTTAAGGATCATTT GATTAACAATTAA
PhCHS	TTAGGTAGCCACACTATGCAGAACCACCGTTTCCACCGTCAGA CCCGGACCAAAGCCGAACAGCACGCCCCATTCCAGGCCTTCA CCCGTGGTGCCCAGACCTTCTTTAGCCGATGCTTTGCGCATTTC ATCCAGAATAAACAGGACGCAGGCAGAGCTCATATTGCCGTA GTTTGACAGAACATTACGGGTCGCTTTCAGTTTTTCCGGTTTCA GACCCAGTTTAATTTCGACCTGGTCCAGGATTGCCGGGCCGCC CGGATGTGCAATCCAGAACAGGCTGTTCCAATCGGAGATACTC AGCGGGCGAAACGCTTCTTCCAGGCTTTTTTCAATGTTTTTGA GATCAGGCCCGGCACATCTTTCAGCAGATGGAACGTCAGACC GACTTCACGCAGGTGACCGTCAATTGCGCCGTGGCTATCCGGC AGCAGGGTTTGAGCTGCGCTGACCAGTTCAAACAGCGGACGTT CAACGCCCGGAATCGGGTCGCTACCAATGATAATGGCACCTG CACCATCACCGAACAGGGCCTGGCCAACCAGGGAGTCCAGGT GCGTATCGTTCGGACCACGAAACGTAACAGCGGTGATTTCACT ACACACCACCAGAACACGCGCACCTTTATTGTTTCGGCCAGA TCTTTCGCCAGACGCAGAACGGTACCGCCTGCGAAGCAGCCTT GCTGGTACATCATCAGGCGTTTCACGCTCGGACGCAGACCCAG CAGTTTCGTCAGTTGATAGTCACAACCCGGCATATCCACACCC GACGTGGTGCAAAAGACCAGATGGGTAATTTTTGATTTCGGTT GGCCCCATTCTTTGATCGCTTTCTGGGCCGCTTCTTTACCCAGTT TCGGCACTTCGACAACCACAATGTCCTGGCGAGCATCCAGAG ACGGTGCCATGTATTCACACATGCTCGGATTTTCTTTCAGAATT TCTTCGGTCAGGTGCATATAGCGTTTTTTGATCATGCTTTTTTCG CACATACGTTTGAATTTTTCTTTCAGATCCGTTTTATGTTCAGAG TTGGTGATACGAAAGTAATAGTCCGGATAGGTGCTCTGATCAA CACAGTTCGTCGGGGTTGCCGTGCCAATCGCCATCACCGTCGC CGGGCCTTCAGCGCGTTGAGCTTTGCGGTATTCTTCCACCGTAA CCAT
MsCHI	ATGGCAGCAAGCATTACGGCAATCACGGTTGAAAATCTGGAA TATCCGGCGGTCGTTACCTCTCCGGTCACGGGCAAATCATACTT TCTGGGCGGTGCCGGTGAACGTGGTCTGACCATTGAGGGTAAC TTTATCAAATTCACGGCAATTGGCGTTTATCTGGAAGATATCGC GGTCGCCTCACTGGCGGCCAAATGGAAAGGTAAAAGCTCTGA AGAACTGCTGGAAACCCTGGATTTTTACCGTGACATTATCTCA GGCCCGTTCGAAAAACTGATCCGTGGTTCGAAAATTCGCGAAC TGAGCGGCCCGGAATATTCTCGCAAAGTCATGGAAAACTGCGT GGCTCATCTGAAATCCGTCGGCACGTACGGTGACGCAGAAGCT GAAGCGATGCAGAAATTTGCCGAAGCATTCAAACCGGTGAAT TTTCCGCCGGGTGCCAGTGTTTTCTATCGTCAATCCCCGGATGG

	CATCCTGGGTCTGTCATTTTCGCCGGACACCAGCATCCCGGAA<br>AAAGAAGCAGCTCTGATTGAAAATAAAGCTGTGAGTTCCGCG<br>GTTCTGGAAACGATGATTGGCGAACACGCGGTTTCTCCGGATC<br>TGAAACGCTGTCTGGCTGCTCGCCTGCCGGCTCTGCTGAATGA<br>AGGTGCCTTTAAAATCGGTAACTGACCTAGG
Pc4CL	ATGGGTGACTGCGTTGCCCCGAAAGAGGATCTGATCTTCCGCA<br>GCAAACTGCCGGACATTTACATTCCAAAGCATCTGCCGCTGCA<br>TACCTATTGTTTTGAGAACATCAGCAAGGTTGGCGACAAGAGC<br>TGTCTGATCAACGGCGCAACCGGCGAAACCTTTACCTACAGCC<br>AGGTTGAGCTGCTGTCCCGTAAAGTTGCCAGCGGCCTGAACAA<br>GCTGGGCATTCAACAAGGTGATACCATTATGCTGCTGCTGCCG<br>AACTCCCCGGAGTACTTTTTCGCTTTCCTGGGTGCGAGCTATCG<br>CGGTGCAATCAGCACTATGGCGAACCCATTCTTTACCAGCGCA<br>GAAGTGATCAAGCAACTGAAAGCGAGCCAAGCGAAGCTGATT<br>ATCACCCAGGCATGCTATGTTGACAAGGTTAAGGACTACGCAG<br>CGGAGAAAAACATCCAGATCATTTGTATTGACGATGCACCGCA<br>GGATTGCCTGCACTTTAGCAAGCTGATGGAAGCGGATGAGAG<br>CGAAATGCCGGAAGTGGTTATTAACAGCGATGATGTGGTGGC<br>ACTGCCGTACAGCTCTGGCACCACCGGCCTGCCGAAAGGCGTT<br>ATGCTGACCCACAAGGGTCTGGTTACCAGCGTTGCACAACAGG<br>TGGATGGTGATAACCCGAACCTGTATATGCACTCCGAGGATGT<br>TATGATCTGCATCCTGCCACTGTTCCATATCTATAGCCTGAACG<br>CTGTTCTGTGTTGTGGTCTGCGTGCGGGCGTTACCATTCTGATC<br>ATGCAAAAGTTCGACATTGTGCCGTTTCTGGAGCTGATTCAGA<br>AGTATAAGGTTACCATTGGTCCGTTTGTTCCGCCGATCGTGCTG<br>GCCATCGCGAAAAGCCCGGTTGTTGACAAGTACGACCTGTCTA<br>GCGTGCGCACCGTTATGAGCGGTGCAGCGCCGCTGGGTAAAG<br>AGCTGGAGGACGCTGTTCGTGCGAAATTCCCGAACGCGAAGC<br>TGGGTCAAGGCTATGGCATGACCGAAGCCGGTCCGGTTCTGGC<br>GATGTGTCTGGCGTTCGCCAAAGAGCCGTATGAGATTAAGTCT<br>GGCGCATGCGGTACCGTTGTGCGTAACGCCGAGATGAAAATC<br>GTTGACCCAGAAACCAACGCGTCTCTGCCGCGTAACCAGCGTG<br>GTGAGATTTGCATCCGTGGTGATCAGATTATGAAAGGTTACCT<br>GAACGACCCGGAAAGCACCCGCACCACCATCGACGAAGAGG<br>GTTGGCTGCACACCGGTGACATTGGTTTCATCGACGATGACGA<br>TGAACTGTTCATTGTTGATCGTCTGAAAGAAATCATTAAGTAC<br>AAAGGTTTTCAAGTTGCTCCGGCGGAGCTGGAAGCACTGCTGC<br>TGACCCACCCGACCATCAGCGATGCCGCGGTGGTTCCGATGAT<br>TGACGAGAAAGCGGGTGAAGTGCCAGTGGCGTTTGTTGTGCGT<br>ACCAACGGTTTTACCACCACCGAAGAAGAAATCAAACAATTT<br>GTGAGCAAACAGGTTGTGTTCTACAAACGTATCTTCCGCGTTTT<br>CTTCGTTGACGCTATTCCGAAATCCCCGAGCGGCAAGATTCTG<br>CGTAAGGATCTGCGCGCTCGTATTGCGAGCGGCGACCTGCCGA<br>AGTAA

GeHIS	ATGTTGGTTGAATTGGCTATCACTTTGTTAGTTATTGCATTGTTT<br>ATTCACTTAAGACCAACATTGTCTGCAAAGTCTAAGTCATTGA<br>GACATTTGCCAAATCCACCATCACCAAAACCAAGATTACCATT<br>CGTTGGTCATTTGCATTTGTTGGATAAGCCATTGTTGCATTACT<br>CTTTGATTGATTTGTCAAAGAGATACGGTCCATTGTACTCTTTG<br>TACTTCGGTTCAATGCCAACTGTTGTTGCTTCTACACCAGAATT<br>GTTTAAATTGTTTTTACAAACTCATGAAGCTTCTTCTTTTAATAC<br>TAGATTCCAAACATCTGCAATCAGAAGATTGACATACGATAAC<br>TCAGTTGCTATGGTTCCATTTGGTCCATACTGGAAGTTTATTAG<br>AAAGTTGATCATGAACGATTTGTTAAATGCTACTACAGTTAAT<br>AAGTTAAGACCATTGAGATCTCAAGAAATCAGAAAGGTTTTA<br>AGAGTTATGGCTCAATCTGCAGAATCACAAGTTCCATTGAACG<br>TTACTGAAGAATTGTTGAAGTGGACTAACTCTACAATCTCAAG<br>AATGATGTTGGGTGAAGCTGAAGAAATTAGAGATATTGCAAG<br>AGATGTTTTGAAGATCTTCGGTGAATACTCTTTGACAGATTTCA<br>TCTGGCCATTGAAGAAATTGAAGGTTGGTCAATACGAAAAGA<br>GAATCGATGATATTTTCAATAGATTTGATCCAGTTATTGAAAG<br>AGTTATTAAGAAAAGACAAGAAATCAGAAAGAAAAGAAAGG<br>AAAGAAATGGTGAAATTGAAGAAGGTGAACAATCAGTTGTTT<br>TCTTGGATACTTTGTTGGATTTCGCTGAAGATGAAACTATGGAA<br>ATTAAAATCACAAAGGAACAAATTAAAGGTTTGGTTGTTGATT<br>TCTTTTCTGCAGGTACTGATTCAACAGCTGTTGCAACAGATTGG<br>GCTTTGTCTGAATTGATTAATAACCCAAGAGTTTTCCAAAAGG<br>CTAGAGAAGAAATCGATGCAGTTGTTGGTAAAGATAGATTGGT<br>TGATGAAGCTGATGTTCAAAATTTGCCATACATCAGATCAATC<br>GTTAAGGAAACTTTTAGAATGCATCCACCATTGCCAGTTGTTA<br>AGAGAAAGTGTGTTCAAGAATGTGAAGTTGATGGTTATGTTAT<br>TCCAGAAGGTGCTTTGATTTTGTTTAATGTTTGGGCAGTTGGTA<br>GAGATCCAAAATACTGGGATAGACCAACAGAATTCAGACCAG<br>AAAGATTTTTAGAAAATGTTGGTGAAGGTGACCAAGCAGTTGA<br>TTTGAGAGGTCAACATTTTCAATTGTTACCATTTGGTTCTGGTA<br>GAAGAATGTGTCCAGGTGTTAATTTGGCTACTGCTGGTATGGCT<br>ACATTGTTGGCATCTGTTATTCAATGTTTCGATTTGTCAGTTGTT<br>GGTCCACAAGGTAAAATCTTGAAGGGTAACGATGCTAAAGTTT<br>CAATGGAAGAAAGAGCTGGTTTAACTGTTCCAAGAGCACATA<br>ATTTGATTTGTGTTCCAGTTGCTAGATCTTCAGCAGTTCCAAAA<br>TTGTTTTCTTCATAA
GmIFS	ATGTTGTTAGAATTAGCTTTGGGTTTATTCGTTTTGGCTTTGTTC<br>TTGCACTTGAGACCAACTCCATCTGCTAAGTCTAAGGCTTTGA<br>GACACTTGCCAAACCCTCCTTCTCCAAAGCCAAGATTACCATT<br>CATCGGTCATTTGCATTTGTTAAAGGATAAGTTGTTGCACTATG<br>CTTTAATTGATTTGTCTAAGAAGCATGGTCCATTGTTCTCATTGT<br>CTTTCGGTTCCATGCCAACTGTTGTTGCTTCCACTCCAGAATTG<br>TTCAAGTTGTTCTTGCAAACTCATGAAGCTACCTCTTTTAACAC

	TAGATTCCAAACCTCTGCTATTAGAAGATTAACCTACGATAAT<br>TCAGTTGCTATGGTTCCATTTGGTCCATACTGGAAGTTTGTTAG<br>AAAGTTAATCATGAATGATTTGCTAAACGCTACTACTGTTAAC<br>AAGTTGAGACCATTAAGAACCCAACAAATTAGAAAATTCTTG<br>AGAGTTATGGCTCAATCAGCTGAAGCTCAAAAGCCATTGGATG<br>TCACTGAAGAACTTTTGAAGTGGACTAATTCTACCATTTCCATG<br>ATGATGTTGGGTGAAGCTGAGGAAATTCGTGATATCGCTAGAG<br>AAGTTTTAAAAATTTTTGGTGAATACTCCTTGACCGATTTTATTT<br>GGCCATTGAAATACCTGAAGGTTGGTAAATACGAAAAAAGAA<br>TTGATGACATTTTGAACAAGTTCGATCCTGTTGTTGAAAGAGTT<br>ATCAAGAAGCGTAGAGAAATTGTTAGGCGTCGTAAGAACGGC<br>GAAGTTGTTGAAGGTGAAGCTTCAGGTGTCTTTTTGGACACATT<br>ATTAGAATTTGCTGAAGATGAAACCATGGAAATCAAGATTACT<br>AAGGAACAAATCAAGGGTCTAGTCGTCGATTTCTTCTCTGCTG<br>GTACTGATTCCACTGCTGTTGCAACTGAATGGGCTCTAGCTGA<br>ACTAATTAACAACCCTAGAGTGTTACAAAAGGCTAGAGAGGA<br>AGTTTACTCTGTCGTTGGGAAGGATCGTTTAGTCGATGAAGTTG<br>ACACTCAAAACTTGCCATATATTAGAGCTATTGTTAAGGAAAC<br>TTTCCGTATGCATCCACCTTTGCCAGTCGTTAAGAGAAAATGTA<br>CAGAAGAATGTGAAATTAATGGTTACGTTATTCCAGAAGGTGC<br>CTTAGTTTTGTTCAATGTTTGGCAAGTAGGTAGAGATCCAAAGT<br>ATTGGGATCGTCCATCTGAATTCAGACCTGAAAGATTTTTAGA<br>AACTGGTGCTGAAGGTGAAGCAGGTCCTTTAGATTTGAGAGGT<br>CAACATTTTCAATTGTTACCATTCGGTTCCGGTAGAAGAATGTG<br>TCCAGGTGTTAACTTAGCTACATCAGGTATGGCTACTTTATTGG<br>CTTCCTTAATTCAATGTTTCGATCTTCAAGTCTTAGGTCCTCAA<br>GGTCAAATCTTGAAAGGCGATGATGCTAAAGTTTCCATGGAAG<br>AAAGAGCCGGTTTGACTGTTCCAAGAGCTCATAGTTTAGTTTGT<br>GTTCCATTAGCTAGAATTGGTGTTGCTTCTAAATTGTTGTCCTA<br>A
GmIFS2	ATGTTGTTGGAATTGGCTTTGGGTTTGTTGGTTTTAGCTTTGTTC<br>TTGCATTTGAGACCAACTCCAACTGCTAAGTCTAAGGCTTTGA<br>GACACTTACCAAATCCACCATCTCCTAAGCCAAGATTACCTTT<br>CATCGGTCACTTGCATTTGTTGAAGGACAAGTTATTGCACTATG<br>CTTTGATTGATTTGTCCAAGAAACATGGTCCATTGTTTCTTTAT<br>ACTTCGGTTCTATGCCAACCGTTGTTGCTTCTACTCCTGAATTGT<br>TTAAATTATTCTTGCAAACACATGAAGCTACCTCTTTCAACACT<br>AGATTCCAAACATCTGCTATTAGAAGATTAACCTACGATTCAT<br>CCGTTGCTATGGTTCCTTTCGGTCCATATTGGAAATTCGTTAGA<br>AAGTTAATTATGAATGACTTGTTAAACGCTACTACCGTCAACA<br>AATTGAGACCATTGAGAACTCAACAAATTCGTAAGTTTTTAAG<br>AGTCATGGCTCAAGGTGCTGAAGCTCAAAAACCATTGGATCTA<br>ACTGAAGAATTGCTAAAATGGACTAATAGTACTATTTCTATGA<br>TGATGTTAGGTGAAGCTGAAGAAATTAGAGACATTGCTAGAG

	AAGTTTTAAAGATTTTCGGTGAATACTCTTTGACCGACTTCATT
	TGGCCATTGAAACACTTAAAAGTTGGTAAATACGAAAAGAGA
	ATTGATGACATCTTAAATAAGTTTGATCCTGTTGTTGAAAGAGT
	TATTAAGAAAAGAAGAGAAATTGTAAGACGTCGTAAAAACGG
	TGAAGTTGTTGAAGGTGAAGTTAGTGGTGTTTTTCTTGATACTT
	TGTTAGAATTCGCTGAAGATGAAACTATGGAAATTAAGATAAC
	TAAGGACCATATTAAGGGTTTAGTTGTTGATTTCTTTTCTGCCG
	GTACAGATTCTACTGCTGTTGCCACAGAATGGGCATTAGCAGA
	ATTGATTAATAACCCAAAGGTTTTGGAAAAGGCAAGAGAAGA
	AGTTTATTCTGTTGTTGGTAAAGATAGATTGGTCGATGAAGTTG
	ATACCCAAAATTTGCCTTACATTAGAGCTATTGTTAAAGAAAC
	TTTTAGAATGCACCCTCCATTACCAGTTGTTAAGAGAAAATGT
	ACTGAAGAATGTGAAATCAATGGTTACGTGATTCCAGAAGGTG
	CTTTGATCTTGTTTAATGTTTGGCAAGTTGGTAGAGACCCTAAA
	TATTGGGATCGTCCTTCTGAATTTCGTCCAGAACGTTTCTTAGA
	AACTGGTGCTGAAGGTGAAGCAGGTCCATTAGATCTAAGAGG
	TCAACATTTTCAATTATTACCATTCGGTTCTGGTAGAAGAATGT
	GTCCAGGTGTTAACTTAGCAACCTCTGGTATGGCTACTTTATTG
	GCTTCTTTGATTCAATGTTTTGACCTACAAGTCTTAGGTCCACA
	AGGTCAAATTTTGAAGGGTGGTGACGCTAAGGTCTCCATGGAA
	GAAAGAGCTGGTTTGACTGTTCCACGTGCTCATTCATTGGTTTG
	TGTCCCATTAGCTAGAATTGGTGTTGCTTCTAAGTTATTGTCTTA
	A
GuIFS	ATGTTGGTTGAATTGGCTATCACCTTGTTGGTCATTGCTTTGTTC
	ATTCACTTGAGACCAACCCCAAGTGCTAAGTCTAAGTCCTTGA
	GACATTTGCCAAACCCACCATCTCCAAAGCCAAGATTGCCATT
	CGTCGGTCACTTGCATTTGTTGGACAAACCATTATTACATAACT
	CTTTGATCGATTTGTCCAAAAGATACGGACCATTGTATTCTCTT
	TACTTTGGTTCTATGCCAACTGTTGTCGTTTCTACTCCTGAATTG
	TTCAAGTTGTTTTTGCAAACTCACGAAGCCTCTTCCTTTAATAC
	CAGATTTCAAACTCCTGCTATTAGAAGATTGACCTACGATAAC
	TCCGTTGCTATGGTCCCATTTGGTCCTTATTGGAAGTTCATTAG
	AAAGTTGATTATGAACGATCTATTGAACGCCACTACCGTTAAC
	AAATTACGTCCATTGAGATCTCAAGAAATTAGAAAGGTTTTGA
	GAGTTATGGCCTTGTCTGCTGAATCTCAAGTTCCTTTGAACGTT
	ACTGAAGAATTATTGAAGTGGACTAATTCTACTATTTCCCGTAT
	GATGTTAGGTGAAGCTGAAGAAATTAGAGATATTGCCAGAGA
	TGTTTTGAAGATTTTTGGTGAATATTCTTTAACTGATTTCATCTG
	GCCATTGAAAAAGTTAAAAGTTGGTCAATACGAAAAGAGAAT
	AGATGATATTTTTAATAGATTTGATCCAGTAATTGAACGTGTAA
	TTAAGAAGAGACAAGAAATCAGAAAAAAGCGTAAGGAAAGA
	AACGGTGAAGTTGAAGAAGGTGAGCAATCCGTCGTTTTTCTTG
	ATACCCTTTTGGATTTCGCTGAAGATGAAACTATGGAAATTAA
	GATTACTAAGGAACAAATCAAAGGTTTGGTTGTTGACTTCTTTT

	CTGCCGGTACTGATTCTACTGCTGTTGCAACAGAATGGGCTTTG<br>TCTGAATTGATTAACAACCCTAGAGTTTTGCAAAAAGCAAGAG<br>AAGAAGTTGACGCTGTTGTTGGTAAAGATCGTTTGGTTGATGA<br>AGCTGATGTTCAAAACTTGCCTTACATTAGATCTATTGTTAAGG<br>AAACCTTCAGAATGCATCCTCCATTGCCAGTTGTTAAGAGAAA<br>ATGTGTTCAAGAATGTGAAATTGATGGTTACGCTATTCCAGAA<br>GGTGCTCTAATTTTGTTCAATGTTTGGGCTGTAGGTAGAGATCC<br>AAAATATTGGGATAGACCTACTGAATTTCGTCCAGAAAGATTT<br>CTAGAAAATGTAGGTGAAGGTGACCAAGCCGTTGATTTGAGA<br>GGTCAACACTTTCAATTATTACCATTTGGTTCTGGTAGAAGAAT<br>GTGTCCAGGTGTTAATTTGGCTACAGCTGGTATGGCTACCTTAT<br>TAGCTTCTGTTATTCAATGTTTTGATTTGTCTGTTGTTGGTCCAC<br>AAGGAAAGATTTTGAAAGGTAATGATGCTAAGGTTTCTATGGA<br>AGAATCCGCCGGTCTAACTGTGCCAAGAGCTCATAACTTAGTT<br>TGTGTTCCAGTCGCTCGTTCTTCTGCTGTCCCAAAGTTGTTCTCT<br>TCATAA
MtIFS	ATGTTGGTTGAATTAGCCGTTACTTTGTTGTTGATTGCTTTGTTC<br>TTACACTTGAGACCAACTCCAACTGCTAAATCTAAAGCTTTAA<br>GACATTTGCCTAACCCTCCATCTCCAAAGCCAAGATTGCCTTTT<br>ATCGGTCACTTGCACTTGTTAGATAATCCATTATTGCATCACAC<br>GCTTATCAAGTTAGGCAAGAGATACGGTCCATTGTACACTTTA<br>TACTTCGGTTCCATGCCAACTGTTGTTGCTTCTACTCCAGATTTG<br>TTTAAGTTGTTCTTACAAACTCACGAAGCTACTTCTTTCAACAC<br>TAGATTCCAAACTAGTGCTATTTCCCGTTTGACTTATGACAATT<br>CTGTTGCTATGGTTCCATTCGCTCCATACTGGAAGTTCATTAGA<br>AAGTTGATTATGAATGATTTGTTAAATGCTACAACTGTTAACA<br>AGTTGAGACCATTGAGATCCAGAGAAATTTTAAAGGTGTTAAA<br>GGTTATGGCTAACTCTGCTGAAACTCAACAACCATTGGATGTT<br>ACTGAAGAATTACTTAAATGGACAAATTCTACTATTTCTACTAT<br>GATGTTGGGTGAAGCTGAAGAAGTTAGAGATATCGCTAGAGA<br>TGTCTTAAAAATCTTTGGTGAATACTCCGTTACTAATTTTATTTG<br>GCCACTAAATAAGTTCAAATTTGGTAACTATGATAAAAGAACT<br>GAAGAAATTTTCAACAAGTATGATCCAATTATTGAAAAGGTTA<br>TCAAGAAGAGACAAGAAATCGTTAACAAAAGAAAGAACGGT<br>GAAATTGTCGAAGGTGAACAAAACGTTGTTTTTCTAGATACTTT<br>GTTAGAATTCGCTCAAGATGAAACCATGGAAATTAAGATTACT<br>AAGGAACAAATCAAGGGTTTGGTCGTGGATTTCTTTTCCGCCG<br>GTACTGATAGCACAGCTGTTTCTACCGAATGGACCTTGTCCGA<br>ATTGATCAACAACCCTAGAGTCTTGAAGAAGGCTAGAGAAGA<br>AATCGACTCAGTCGTAGGTAAAGATAGATTAGTTGACGAATCT<br>GATGTTCAAAACTTACCTTATATTAAGGCTATTGTCAAGGAAG<br>CCTTCAGATTGCATCCTCCATTACCAGTTGTCAAAAGAAAGTG<br>TACCCAAGAATGTGAGATTGACGGTTACGTTGTGCCAGAAGGT<br>GCTTTGATTTTGTTCAATGTCTGGGCTGTTGGACGTGATCCAAA

	GTACTGGGTTAAACCATTGGAATTCAGACCTGAACGTTTCATT GAAAATGTTGGTGAAGGTGAAGCTGCCTCTATTGATTTGAGAG GTCAACATTTCACATTATTACCATTTGGTTCTGGTAGAAGAATG TGTCCAGGTGTTAACTTGGCCACTGCTGGTATGGCCACAATGA TTGCTTCTATCATCCAATGTTTTGATTTGCAAGTTCCAGGTCAA CATGGTGAAATTTTGAACGGTGACTACGCTAAGGTTTCTATGG AAGAAAGACCAGGTTTGACTGTTCCAAGAGCTCATAACTTGAT GTGTGTTCCATTGGCTAGAGCTGGTGTTGCTGATAAGTTGTTAT CTTCCTAA
PlIFS	ATGTTATTAGAATTAGCTTTAGGTTTGTTAGTTTTAGCTTTGTTT TTACACTTGAGACCAACCCCAACTGCTAAATCTAAAGCCTTGA GACATTTGTTGAATCCACCATCTCCAAAGCCAAGACTACCATT CATCGGTCATTTGCACTTATTGAAGGATCAATTGTTGCACCATG CTTTGATCGATTTGTCTAAGAAGTATGGTCCATTGTACTCTTTGT ATTTCGGTTCTATGCCAACTGTTGTTGCCTCTACTCCAGAACTA TTCAAGCTTTTCTTGCAAACTCATGAAGCAACCTCCTTTAATAC TAGATTTCAAACTTCAGCTATTAGAAGATTAACTTACGATAAC TCTGTCGCTATGGTCCCATTCGGTCCATACTGGAAGTTCATTAG AAAGTTGATTATGAACGATTTGCTTAATGCTACTACCGTTAATA AGTTACGTCCATTGCGTACTGAACAAATTAGAAAGTTTCTAAG AGTTATGGCCCAATCCGCTGAAGCTCAACAACCATTGAACGTT ACTGAAGAATTGTTAAAATGGACTAATTCTACTATCAGTATGA TGATGTTAGGTGAAGCTGAAGAAATTAGAGATATAGCTAGAG AAGTCTTGAAAATCTTCGGTGAATATTCTGTTACAAACTTTATT TGGCCATTGATGTATTTGAAGGTTGGTAAATACGAAAAGAGAA TCGATGATATTTTTAACAAGTTCGATCCAGTTATTGAAAGAGTT ATCAAAAAGAGAAGAGAAATTGTTAGACGTAGAAAGAACGGT GAAGTCGTTGAAGGTGAAGCTTCCGGTGTTTTCTTAGATACTTT ATTGGAATTCGCTGAAGATGAAACTATGGAAATCAAGATAACT AAGGAACAAATTAAGGGTTTAGTTGTTGATTTCTTTTCTGCAGG TACTGATTCTACTGCTGTTGCTACTGAATGGGCCTTAGCTGAAT TGATTAATAACCCAAGAGTTCTTCAAAAAGCTAGAGATGAAGT TTACTCCGTTGTCGGTAAAGATAGATTAGTTGATGAAGTTGAC ACTCAAAACTTGCCATACATTAGAGCTATTGTTAAGGAAACAT TCAGAATGCACCCACCATTGCCTGTTGTTAAGAGAAAGTGTGT TGAAGAATGCGAAGTTAATGGTTTTGTCATCCCTGAAGGTGCT TTAATCTTGTTTAACGTCTGGGCTGTTGGTAGAGATCCTAAATA CTGGGATAGACCACTAGAATTCAGGCCAGAAAGATTCTTAGA ATCCGGTGCTGAGGGTGAAGTCGGTCCAATTGACTTGAGAGGT CAACATTTCCAATTGTTACCATTCGGTTCTGGTAGACGTATGTG TCCAGGTGTTAATTTGGCCACATCTGGTATGGCTACATTGTTGG CTTCCTTGATTCAATGTTTTGATTTGCAAGTAGTTGGTCCAAAA GGTGAAATTTTGAAGGGAAAAGATGCCAAGGTCTCCATGGAA GAATCCGCTGGTTTGACTGTTCCTAGAGCTCACAACTTGAGAT

	GTGTTCCATTGGCTAGAACCTCCGTCGCTGCTAAGGTTTTGTCT TAA
TpIFS	ATGTTGTTGGAATTGGCCTTGGGTTTGTTGGTCTTGGCTTTGTTC TTGCACTTGAGACCAACTCCAACTGCTAAATCTAAAGCTTTGA GACATTTGCCAAACCCACCATCTCCAAAACCTAGATTGCCATT CATTGGTCATTTGCATTTGTTGAAGGATAAGTTGTTGCATTACG CTTTGATTGATTTGTCTAAGAAACATGGTCCATTATTTTCATTAT ATTTCGGTTCTATGCCAACTGTCGTTGCTTCTACTCCTGAATTGT TTAAATTATTCTTACAAACCCATGAAGCTACTAGTTTTAACACT AGATTTCAAACTTCAGCTATTAGAAGATTAACTTATGATTCTTC TGTTGCCATGGTTCCAATTGGTCCATATTGGAAATTCGTTAGAA AGTTAATTATGAATGACTTGTTGAACGCTACTACTGTTAACAA GTTAAGACCATTAAGAACGCAACAAATTAGAAAGTTTTTAAG AGTTATGGCACAAGGTGCTGAAGCTCAAAAACCACTAGATTTG ACTGAAGAATTGTTAAAATGGACCAATTCTACCATTTCCATGA TGATGTTAGGTGAAGCTGAAGAAATTAGAGATATTGCTAGAGA AGTTTTAAAGATCTTCGGTGAATATTCCTTGACTGATTTCATTT GGCCTCTTAAACATTTGAAGGTTGGCAAGTACGAAAAGAGAA TTGACGATATTTTAAACAAGTTTGATCCAGTTGTTGAAAGAGTT ATTAAGAAGCGTAGAGAAATTGTTAGACGTCGTAAGAACGGT GAAGTTGATGAAGGTGAAGTTTCTGGTGTTTTCTTGGATACTTT GTTAGAATTTGCTGAAGATGAAACTACTGAAATCAAAATCACA AAGGATCATATTAAGGGTTTAGTTGTTGACTTTTTCTCTGCTGG TACTGATTCTACCGCTGTTGCTACTGAATGGGCTTTAGCTGAAT TAATTAATAACCCTAAAGTTTTAGAAAAAGCTAGAGAGGAAG TTTATTCTGTTGTTGGTAAAGATAGATTAGTTGATGAAGTTGAT ACTCAAAACTTGCCATACATTAGAGCCATCGTCAAGGAAACTT TTAGAATGCATCCACCATTGCCTGTCGTTAAGAGAAAATGTAC TGAAGAATGTGAAATCAATGGTTACGTCATTCCAGAAGGTGCA TTGATTTTGTTCAACGTTTGGCAAGTTGGTAGAGATCCTAAATA CTGGGATAGACCAAGTGAATTTCGTCCTGAAAGATTTTTAGAA ACTGGCGCTGAAGGTGAAGCTCGTCCATTGGACTTAAGAGGTC AACATTTTCAATTGTTACCTTTTGGTTCTGGTAGAAGAATGTGC CCAGGTGTTAACTTGGCTACTTCTGGAATGGCTACTTTGTTGGC TTCTTTAATTCAATGTTTTGATTTGCAAGTTTTAGGTCCACAAG GTCAAATTTTGAAAGGTGGTGACGCTAAAGTTAGTATGGAAGA AAGAGCAGGTTTGACTGTTCCAAGAGCTCATTCTTTGGTTTGTG TTCCATTGGCTAGAATTGGTGTTGCTTCTAAGTTGTTGTCTTAA
GmHID	ATGGCTAAGGAAATCGTTAAGGAATTGTTGCCATTGATCAGAG TTTACAAGGATGGTTCTGTTGAAAGATTGTTGTCTTCAGAAAAC GTTGCTGCATCACCAGAAGATCCACAAACTGGTGTTTCTTCAA AGGATATCGTTATCGCTGATAACCCATACGTTTCTGCAAGAAT TTTCTTGCCAAAATCACATCATACAAACAATAAGTTGCCAATT TTCTTGTACTTTCATGGTGGTGCTTTCTGTGTTGAATCTGCTTTTT

	CTTTCTTTGTTCATAGATACTTAAACATCTTGGCTTCTGAAGCA
	AACATCATCGCTATCTCAGTTGATTTCAGATTGTTGCCACATCA
	TCCAATTCCAGCTGCATATGAAGATGGTTGGACTACATTGAAG
	TGGATCGCTTCTCATGCAAACAACACTAACACTACAAATCCAG
	AACCATGGTTGTTGAACCATGCTGATTTCACTAAGGTTTACGTT
	GGTGGTGAAACATCTGGTGCTAACATCGCACATAATTTGTTGTT
	GAGAGCTGGTAACGAATCATTACCTGGTGACTTGAAAATTTTG
	GGTGGTTTATTGTGTTGTCCATTTTTCTGGGGTTCTAAACCAATT
	GGTTCAGAAGCTGTTGAAGGTCATGAACAATCTTTGGCAATGA
	AAGTTTGGAATTTTGCTTGTCCAGATGCACCAGGTGGTATTGAT
	AATCCATGGATTAATCCATGTGTTCCAGGTGCTCCATCTTTGGC
	TACTTTGGCATGTTCAAAGTTGTTGGTTACTATCACTGGTAAAG
	ATGAATTCAGAGATAGAGATATCTTGTACCATCATACAGTTGA
	ACAATCTGGTTGGCAGGGTGAATTACAATTGTTTGATGCTGGT
	GACGAAGAACATGCATTCCAATTGTTTAAACCAGAAACACATT
	TGGCTAAGGCAATGATCAAGAGATTAGCTTCATTTTGGTTTAA
GeHID	ATGGCTTCTTCTACTTCTACTACCACTTCTAAAGAAATTGATAG
	AGAATTACCACCATTGTTGAGAGTTTACAAGGATGGTACCGTT
	GAAAGATTCTTGGGTTCCTCTTTTGTTCCTCCTTCTCCAGAAGA
	TCCAGAAACCGGTGTTTCTACTAAGGATATCGTCATTTCTGAA
	AACCCAACTATCTCTGCTAGAGTTTACTTGCCAAAGTTAAACA
	ACACTACTGAAAAGTTGCCAATTTTAGTTTATTACCACGGTGGT
	GCTTTTTGTTTAGAATCTGCTTTTTCTTTCCTTCATCAAAGATAC
	TTAAACATTGTTGCTTCTAAAGCTAACGTTTTGGTAGTCTCTATT
	GAATATAGATTAGCTCCAGAACATCCATTGCCAGCAGCTTACG
	AAGATGGTTGGTACGCTTTGAAGTGGGTTACCTCTCACTCTACC
	AACAACAACAAGCCTACTAACGCTGATCCATGGTTGATTAAGC
	ACGGCGATTTCAACAGATTTTACATTGGTGGCGACACCTCTGG
	TGCTAATATTGCCCATAATGCCGCCTTGAGAGTTGGTGCTGAA
	GCTTTGCCAGGTGGTTTAAGAATTGCTGGTGTTTTATCTGCTTTC
	CCATTGTTTTGGGGTTCCAAGCCTGTTTTGTCCGAACCAGTTGA
	AGGTCATGAAAAGTCCTCCCCAATGCAAGTTTGGAATTTCGTT
	TATCCAGATGCTCCAGGTGGTATTGATAATCCATTGATTAACCC
	ATTGGCTCCAGGTGCTCCAAACTTGGCTACATTGGGTTGTCCA
	AAGATGTTGGTTTTCGTCGCTGGTAAAGATGACTTGAGAGATA
	GAGGTATTTGGTACTACGAAGCTGTTAAGGAATCTGGTTGGAA
	GGGTGACGTTGAATTGGCTCAATATGAAGGTGAAGAACATTGT
	TTCCAAATCTACCATCCAGAAACTGAAAACTCTAAGGATTTGA
	TTGGTAGAATTGCTTCTTTCTTGGTTTAA
PlHID	ATGGCTAACGAAAACTCTAATAAGGAAATTGTTAAGGAAGTTT
	TGCCATTGATTAGAGTTTACAAGGATGGTACTGTTGAAAGATT
	ATTGTCCTCTCCAAACGTTGCTGCTTCTCCAGAAGATCCAGAA
	ACTGGTGTTAGTTCTAAGGATATTGTTATTGCTCATAACCCATA
	TGTTTCTGCTAGAATCTTCTTGCCTAACATCAATAAGTCTCATA

	ACAAGTTGCCAATCTTCGTTTACTTTCATGGTGGTGCTTTCTGTG TTGAATCTGCTTTCTCTTTCTTTGTTCATAGATACTTGAATATTTT AGCTTCTCAAGCTAATATTATCGCCGTTTCTGTTGATTTCAGATT GTTGCCACATCATCCTTTGCCAGCCGCTTATGAAGATGGTTGG ACTACTTTGCAATGGATCGCCTCACATGCTAACAACACTGCTA CTAATCCAGAACCATGGCTATTGAACCATGCTGATTTTAACAA GTTGTACGTTGGTGGTGAAACTTCTGGTGCTAATTTGGCTCATA ACTTGCTATTGCGTGCTGGTAACGGTAATCAATCCTTGCCAGGT GACTTAAAGATTTTGGGTGGTTTGTTGTGTTGTCCATTCTTCTGG GGTTCTAAGCCAATCGGTTCTGAACCAGTTGATGAACATGAAC AATCCTTAGCTATGAAGGTTTGGAACTTAGCTTGTCCAGATGCT CCAGGTGGTATTGATAATCCATGGATTAATCCTTGTGTTGCTGG TGCTCCTTCTTTAGCCACCTTGGGTTGTTCTAAGTTGTTAGTTAC TATTACTGGTAGAGATGAGTTTAGAGATAGAGATATTTTGTATC ATGATACCGTTAAAAAATCAGGTTGGGAAGGTCAATTGGAATT GTTCGACGCCGGGGATGAAGAACATGCCTTCCAATTGTTCAAA CCAGAAACTGATACTGCTAAGGCTATGATCAAGAGATTAGCTT CCTTCTTGGTTTAA
GmCPR	TTACCAAACATCTCTCAAGTATCTACCAGTAGTTTGCAAGTTCT TAACCATAGATTCAGCTTTACTACTATCTAGTGAACCTTGTTCT TGCAAGATAGTATGTAGGGCTCTATGAACATCACGTGCCATAC CCTTAGCATCTCCACAAACGTAGATGTAAGCACCTTGAGAAAT CATGGACCAAATTTCAGAAGCCTTTTCCATCATCTTGTGTTGGA CGTATTCCTTTGTTGGACCTTCTCTGCTGAAAGCTAAGATCAAT TCAGATAAAGCACCTGTATTGACGAAATGGTTCAATTCATCTT CATAGATATAATCCATTTGTCTATTTCTACAACCGAAGAATAA AACAGAAGGACCCAATTCAGCACCACCACCCTTTAAGGCCAA TCTTTCTTGTAAGAAACCCCTAAAAGGGGCCAAACCGGTACCT GGACCAATCATAATGATTGGAACCTTATTATCTGCTGGTAATCT GAAATTGCTAGTACGGACGAAAATTGGAGCCCAAGAGCAGTC TTGGGATTTTTCTAATGGGACAGAGTTCTTCATCCAAGTAGAAC AAACACCCTTATGAATTCTACCAGTTGGCATCTTATCATGAACT AAAGCGCAGGTAACATGAATTCTGTTAGGAACCATTCTTGGGG AGGAAGAGATAGAATAAAATCTAGGTTGTAACCTAGGTGCGA CGGCGGCGAAAAAGACTCCAATTGGTGGCTTAGCTGATGGGA ACTCAGCCATAACTTCCAATAATGATCTTTGGGAAGTGATGAC CCATTCGGAGTATTCATCTTTACCAGCCGGGGAAGCCAAATGT CTCAATCTATCAGCTTCAGATGGATCAGAAGCATGAGCAGCCA AGGCTAATAAAGCAGACTTTTTAGGAGAAGATAAAACGTCAG CGTATTGAGCTAAAGCTTTACGTAAAGTACATGGTGGGAATGT TGGTGGTAAGGAAGAACCACCTAATGGCTTGCCATCTTCATCA TCAGTATGAATAGAGAAGTACGTGTCTGGGGATAAACCAATC AATCTAATAGCTTCTTCAACGGTTTCACTTAAGTTTTCACAGTA AACACCGACATGGTCACCGGTTTCGTAAGTAACACCGGTACCG

	GAAATGTCAAATTCTAAGTGAGTACATGATCTATCTGAAGCGG GGGTGTGCAATTCTTTTCTAACAGCAACGTTAGCACGAACTGG ATGTTGAGCGTCAACAATGGCGTGACCGTTAACATTATGCCAT TTCTTTTCATCAACTGAAGCTTCCAATGGATCATGAATAACGAC TCTGTATTCTAAGACAGCAGCGGTGTATGGAGTGGAAACAGTA GTATCATCTTCATCTCTTAGCAAATCGTCCAAGGCTGGCCACA ATTCTTCCTTCCAAGCGGTAAAATCATCTTCAATACATTGGTCA TCATCTCCTAAACCAACTGGAACTAATCTTTTACCACCTTGTTC AGCCAACATATCATCTACGACCTTGGCAACCTTGTTAAAGTGT TCATATTGACGATTACCTAGACCGAAAACACCATAGTGCAAGT TTTCTAACCAACCTTCACCCTTTTCACCACCTTCAGTGAACCAC TTGTAGAAACGAGCAGCATTATCAGTGGGTTCACCGTCACCGT AGGTGGCTAAGAAAAATAAAGCGTGAGTTTCTTTTTTGAAACG TTCTTCATATTCATCATCATCAGCAGCGTAATCATCCATATCAA CCACTCTAAAAGTGGCTTTATCATATCTAGCCTTAGCTTCTTCA GCTATAGCTTTAGCGAAACCTTCAGCTGTTCCAGTTTGAGTACC GAAGAAAATAGTAACTTTTTTAGTACCATCATCAACCTCAATTT CAGGTAATTTTTCAATAACTCTCTTTGGTGGTTCTAAAGGTTTA GCTTTTGGAGAGGAAGATCTTCTCCAAATAAAAACAACGAAA CAACCAACCAAAACGGCTACAGAAGTAGTCAATAACATTAAG AATTGTCTATTTTCTAACAAAACGGCAGCAGCTGCATTAGAAG AAGAAGATGAAACGTTAGATGGATCCAATTTACCCTTAATAAT AGCAGACATCAAATCTAATGGGGAAATCTTCATGGAACCAGA ATCTTGCAT
CrCPR2	TTACCAAACATCTCTTAAGTATCTACCTGTCATTTGCAAGTTCT TAACGAAACCTTCAGCTTGAGTTGAATCCATAGAACCTTGTTCT TGTGCAATTGTATGCAAAGTTCTATGAACATCTCTTGCCATACC CTTAGCATCACCACAAACGTAAACATAAGCACCATCAGAGAT CATTCTCCAGATATCTGAAGCCTTTTCTGCCATCTTATGTTGAA CGTATTGCTTAGTTGGACCTTCTCTAGAGAATGCAACCAACAA TTCTGACAAAGCACCGATTTCCAAGAAATGGTTCAATTCATCTT CGTAGATGTAATCCATCTTTCTGTTTCTACAACCGAAAAAGAA AACTGCTGTACCCAATTCAGCACCTTCTTCTTTTAAAGCCAATC TTTCTTGCAAGAAACCTCTAAATGGTGCCAAACCAGTACCTGG ACCAATCATAATAACTGGAACCTTTGGATCAGCTGGCAACTTG AAGTTAGATTGTCTAACGAAGATTGGAGCCCATGAACAATCTC TAGATTCTTCCAATGGAATTGCATTTTTCATCCAAGTTGAACAA ACACCTTTATGAATTCTACCACCTGGTGTTTTTTCGTAAACCAA AGCACAAGTAACATGAATTCTAGATGGTGCCATTCTTGGTGAA GATGAGATTGAGTAGAATCTTGGTTGCAATCTTGGAGCAATTG CAGCAAAGAAAACACCCAATGGTGGTTTTGCAGATGGAAATT CAGCCATAACTTCCAACAAAGATCTTTGGTTTGCAACCAATGA TTGAGCGTATTCATCTTTACCAGCTGGTGATGCTAAGTACTTCA ATCTATCTGCTTCGTTTGGATCAGAAGCGTATGCAGCCAAAGC

	TAACAATGCAGATTTTTTAGGTGTGTTCAACAAATCTGCGTATC<br>TTGTCAAAGCAGTTCTCAATGTACATGGTGGAAATGGTGGTGG<br>TAAAGATGAACCAGCCAATGGTGTACCATCTTCTTTATCTGCAT<br>GCAAAGAGAAGTAAGTTTCTGGTGGCAAGTTCAACAATCTTTC<br>AGCTTCTTCAACAGTTTCAGACAAGTTATCACAGTAAACACCA<br>ACATGATCACCTGTACCATATGATAAACCAGTACCAGCAATAT<br>CGAAATCCAAATGTGTACATGATCTATCAGAAGCTGGAGTATG<br>CAATTCCTTTCTAACTGCAACGTTAGATCTACATGGATGTTGAG<br>CATCGTAAACTGTGTTACCGTTTGCGTAACCGTTAGCATGACC<br>ATTTGCTTCAGAGATCAATGAATCAGACTTATCAGGGAAAACA<br>ACTCTGTATTCTGGAATTGCAGCAGTGTATGTAGTTGAAACTGT<br>AGTATCATCTTCATCTCTCAACAAGTTATCCAATTCTGGCCAAA<br>CATTTTCTCTCCATGCAGCAAAATCATCTTCGATACATTGATCA<br>TCATCACCTAAAACCAATGGAACAATTCTTTTACCACCTTGTTC<br>TGCAACTTTTTCATCAACAACTTTAGCAATCTTGTTGAAATGTT<br>CGTATTGTCTGTTGCCTAAACCGAAAACACCGTATTGCAAGTT<br>CTTCAACCAATCACCACGATCGTTACCCTCAACGAACCACTTG<br>TAGAATCTTGCAGCATTATCTGTTGGTTCACCATCACCATAAGT<br>TGCCAAGATAAAGAAAGCCAATGTTTCCTTTCTGAACTTTTCTT<br>CGTATTCTTCATCATCTGCAGCATAATCATCGATATCGATAACC<br>TTGATAACAGCTTTTTCATATCTAGCTTTTGCTTCTTCAGCCAAT<br>GCTTTAGCAAAACCTTCTGCAGTACCTGTTTGAGTACCAAAGA<br>AAATAGTGAATTTTTTCTTACCTTCATCGATTTCTTCTGGTTCAA<br>CAACAGACTTTGGAACGATCAACTTTGGTGGTTCAACAACTTT<br>TTTACCTGAACCAGATGATCTTCTCCAGATCAAAACAACAACA<br>CAACCGATCAAAACAGCAACAGATGTAGTCAAGATCATAACC<br>AATTCCTTGTTTTCCAACAACATAGCCATAACTGCTGGAGAAA<br>CAGCAACACCTGAATCAGATGAGTTGGAACCATCCAACTTTGC<br>ACCTTTCAAGATAGCAGACATCAATTCGAATGGTGACAACTTT<br>TCAGATGAAGAATCCAT
AtCPR	TTACCAAACGTCACGCAGGTAACGGCCAGAGGTCTGCAGGTTT<br>TTCACGAAACCTTCCGCTTTGGTAGAATCCATAGAACCCTGTTC<br>TTGCGCGATGGTGTGCAGAGAACGGTGAACATCACGCGCCAT<br>ACCTTTTGCATCGCCACAAACGTACAGGTACGCGCCCTGAGAA<br>ATCATGTTCCAGATATCGCTTGCTTTATCCATCATTTTGTGCTGA<br>ACATATTCTTTGGTCGGACCTTCACGGCTGAACGCGACGGACA<br>GTTCGGCCAGCGCACCGCTTTCAACAAAGCGCTGCAGTTCTTC<br>TTCGTAGATGAAATCCATACGGCGGTTACGGCAGCCGAAGAA<br>CAGAACGGACGGACCCAGTTCAACGCCAGATTCAACCAGCGC<br>CAGACGTTCCTGCAGGAAGCCACGGAACGGAGCCAGACCGGT<br>ACCCGGACCGATCATAATAATCGGAACTTTAGAATCAGACGG<br>CAGTTTAAAGTTGCTCTGGCGCACGAAGATCGGCGCGGAGGA<br>GCAGTTTTCAGATTTTTCGTATGGAACCGCGTTTTTCATCCAGG<br>TGCTACATACGCCTTTGTGGATACGACCAGTCGGCATTTTTTCA

	TACACCAGCGCGCAAGTAACGTGAATGCGGGTTTCAGCGATTT<br>TCGGGCTGCTAGAGATGGAATAGAAACGCGGCTGAAGGCGCG<br>GAGCCACGCCAGCGAAAAACACGCCCAGCGGCGGTTTGGCGC<br>TCGGGAATTCCGCCATAACTTCCAGCAGAGAGCGCTGGCTTTC<br>CACCACCCATTTAGAGTATTCGTCTTTGCCTGCCGGAGAGGCC<br>AGGTGTTTCAGACGTTCGGCTTCGGTTGGGTCGGATGCGTGCG<br>CCGCCAGCGCTACCAGCGCGGATTTTTTCGGGGAGCTCAGCAG<br>GCATGCGTAGCGGGTCAGCGCAGTGCGCAGGTTGCACGGCGG<br>GAACGGTGGCGGGAGGGAGGATGAGATCGGGGTACCATCTTC<br>TTTTTCAGCATGCAGACTGAAATAGGTATCCGGAGACATGTCC<br>AGCAAACGCAGAGCTTCATCAACGGTTTCAGACAGGTTGTCAC<br>ACAGAACGCCAACGTGGTCACCGGTTTCATAGGTCAGACCGCT<br>GCCCGCAATGTCGAATTCCAGGTGGATACAGGAGCGGTCAGA<br>TTCTGGGGTATGCAGTTCACGTTTAACGGCCACGTTCGCTTTGT<br>ACGGATGCTGTGCGTCGAACACAGTGTAGCCGTTGCCGTTCGC<br>CATGTTAATGTCGTTGAATTTAGCATCTTCGCTATCATGGATGC<br>TAACACGGTATTCCAGAACCGCAGCGGTATACGGGGTTGCCAC<br>GGCGGTATCACCTTCTTCACGCAGGATCGTATCCAGTTCCGGC<br>CACAGCGCTTCGCGCCAAGCGGTGAAGTCATCTTCGATGCACT<br>GGTCATCATCACCCAGGCCTACCTGAACCAGACGCTGTGCGCC<br>CTGTTCAACCAGGATATCGTCTACCACTTTCGCAACTTTGTTGA<br>AGTGTTCATACTGGCGGTTACCCAGGCCAAACACACCGTATTT<br>CAGGTTTTTCAGCCATTCACCACGATCGTTACCTTCAGTGAACC<br>ATTTATAGAAACGAGCCGCGTTATCAGTCGGTTCGCCGTCACC<br>GTAGGTAGCCAGGAAGAAAAACGCAACATCTTCTTTTTTCAGT<br>TTTTCTTCATATTCATCATCATCTGCCGCGTAATCGTCCAAGTC<br>AACGATTTTGAAGCGGGTTTTTTCATAACGAGCTTTGGCTTCTT<br>CACCCAGCGCTTTCGCGAAACCTTCCGCCGTGCCGGTCTGAGT<br>GCCGAAGAAGATGGTAACTTTTTTACGGCCATCATCGATTTCCT<br>CTTCACGCGGTTTGATCACCAGCGGTTTCAACGGTTCAACACG<br>TTTGGAGTTACCGCTACCGCTACGGCGCCAAACCAGCATAACG<br>ATGCAACCGATCAGAACCGCGATGCTGGTGGTCACGATCATA<br>GCGAACTGACGGTTTTCGATCAGCATAGAGCTCAGTTCAGCCG<br>CTACAGATTCGTATGCGCTCGCGTTAGCCGGGTCAGACACGAT<br>CACCGGTTCACCTTTGATGATCGCCGCCATCAGATCAATCATG<br>CTGGTAGAAGAAGAAGAGCTAGAGCTCAT
LjCPR	TTACCAGACATCCCTCAAGTACCTACCGTTCAATTGCAGGTTCT<br>TGACCATACCTTCAGCCTTAGAAGAGTCCAAAGAACCCTGTTC<br>TTGCAGGATAGTGTGCAAGGTTCTGTGAACATCTCTAGCCATA<br>CCCTTAGCATCACCACAGACGTAGATGTAAGCACCTTGGGAGA<br>TCATGTTCCAGATGTCAGAAGCCTTTTCCATCATCTTGTGCTGG<br>ACGTATTCTTTAGTTGGACCTTCCCTGGAGAAGGCAACGATCA<br>ATTCAGACAAAGCACCAGAGTTAACGAAATGGTTCAATTCATC<br>TTCGTAGATGTAATCCATTTGTCTGTTTCTACAGCCGAAGAACA

	AAACAGATGGACCCAATTCAGCACCATCCTCCTTCAAAGCCA GTCTCTCTTGCAGAAAACCTCTGAATGGAGCCAAACCAGTGCC TGGACCGATCATGATAATTGGAACCTTGTTGTCGGCTGGCAAC TTGAAGTTAGATTGTCTGACGAAGATTGGAGCCCAAGAACAAT CTTGGGACTTCTCCAATGGGACAGAGTTCTTCATCCATGTGGA ACAGACACCTCTATGGATTCTACCTGTTGGCATTTTGTCGTTGA CCAGAGCACAGGTAACATGAATCCTAGATGGGGCCATTCTTGG AGAAGAAGAGATGGAGTAAAATCTTGGTTGCAGTCTTGGAGC GATAGCAGCGAAAAAAACGCCAATTGGTGGCTTAGCAGATGG AAATTCGGCCATAACCTCCAGCAATGATCTTTGAGAGGCGATA ACCCACTCAGAGTACTCATCTTTACCAGCTGGAGAAGCCAAAT GCCTCAACCTATCAGCTTCAGATGGGTTAGAAGCATGAGCAGC CAAAGCCAACAAGACAGACTTCTTTGGGGAGGACAAAACATC AGCGTATCTAGCGATAGCGGTTCTCAAAGTACATGGTGGGAAA GTTGGTGGCAAAGAAGAGCCAGACAATGGCTTACCATCTTCAT CATCAGTATGAACAGAGAAGTAAGTATCTGGGGATAAACCCA ACAGTCTGACAGCTTCTTCGACTGTTTCAGACAGGTTCTCGCAG TAAACACCAACATGATCACCAGTCTCGTAAGCAACACCAGTA CCAGAGATATCGAACTCCAGATGGGTGCATGATCTATCAGAA ACTGGGGTATGCAGCTCCTTTCTGACAGCAACATTGGATCTGA CTGGATGTTGAGCGTCAACAATAGCATGGCCGTTAACGTTGTG CCACTTCTTTTCATCAACAGAAGCGTCTAATGGGTCGTGGATA ACGACTCTGTACTCCAAAACAGCAGCAGTGTATGGTGTAGAG ACTGTAGTGTCGTCATCACCTCTCAACAACTCATCCAAAGCTG GCCACAATTCTTCCTTCCAAGCAGTGAAATCATCTTCGATACAT TGATCATCGTCACCCAAGCCAACCTTAACCAGTCTTTTACCAC CGAAATCGGCCAACTTGTCATCAACTTCGATAGCAACCTTGTT GAAATGTTCGTATTGTCTATTACCCAAGCCGAAAACGGCGTAT TCCAAGTTTCTCAACCAACCTTCTTCCTTTTCGTCACCTTCCAGG AACCACTTGTAGAATCTAGCAGCGTTATCAGTTGGTTCACCAT CGCCGTAAGTAGCCAAGAAGAACAAAGCCAAAGTTTCCTTCTT CAACTTTTCTTCGTATTCATCATCATCTTGAGCGTAATCATCCAT ATCAACGATCTTGAACTTAGCTTTCTCGTACCTAACCTTGGCTT CTTCAGCAATAGCCTTAGCAAAACCTTCGGCAGTACCAGTTTG AGTACCGAAGAAGATAGTAACCTTTCTAGTACCATCATCAACT TCAGCTTCATCAGACAACTTTTCAACAACTCTTTTTGGTGGTTC GATGGACTTGGCCTTGTTACCAGTTGATCTTCTCCAGATGAAAA CAACAACACAACCGATCAAAACAGCGATAGAAGTAGTCAAA ACCATAACAAATTCTCTGTTTTCCAAAAAAACAGAACCAGCAC CGGAGGTAGAAGAAACGTTAGATGGGTCCAAGGTGCCCTTAA TCATAGCAGACATCAGATCCAGGGGACTGATCTTCATAGAAG AGGATTCCTCCAT
EcPntAB	TGGAAGGGAATATCATGCGAATTGGCATACCAAGAGAACGGT TAACCAATGAAACCCGTGTTGCAGCAACGCCAAAAACAGTGG

AACAGCTGCTGAAACTGGGTTTTACCGTCGCGGTAGAGAGCGG
CGCGGGTCAACTGGCAAGTTTTGACGATAAAGCGTTTGTGCAA
GCGGGCGCTGAAATTGTAGAAGGGAATAGCGTCTGGCAGTCA
GAGATCATTCTGAAGGTCAATGCGCCGTTAGATGATGAAATTG
CGTTACTGAATCCTGGGACAACGCTGGTGAGTTTTATCTGGCCT
GCGCAGAATCCGGAATTAATGCAAAAACTTGCGGAACGTAAC
GTGACCGTGATGGCGATGGACTCTGTGCCGCGTATCTCACGCG
CACAATCGCTGGACGCACTAAGCTCGATGGCGAACATCGCCG
GTTATCGCGCCATTGTTGAAGCGGCACATGAATTTGGGCGCTT
CTTTACCGGGCAAATTACTGCGGCCGGGAAAGTGCCACCGGC
AAAAGTGATGGTGATTGGTGCGGGTGTTGCAGGTCTGGCCGCC
ATTGGCGCAGCAAACAGTCTCGGCGCGATTGTGCGTGCATTCG
ACACCCGCCCGGAAGTGAAAGAACAAGTTCAAAGTATGGGCG
CGGAATTCCTCGAGCTGGATTTTAAAGAGGAAGCTGGCAGCG
GCGATGGCTATGCCAAAGTGATGTCGGACGCGTTCATCAAAGC
GGAAATGGAACTCTTTGCCGCCCAGGCAAAAGAGGTCGATAT
CATTGTCACCACCGCGCTTATTCCAGGCAAACCAGCGCCGAAG
CTAATTACCCGTGAAATGGTTGACTCCATGAAGGCGGGCAGTG
TGATTGTCGACCTGGCAGCCCAAAACGGCGGCAACTGTGAAT
ACACCGTGCCGGGTGAAATCTTCACTACGGAAAATGGTGTCAA
AGTGATTGGTTATACCGATCTTCCGGGCCGTCTGCCGACGCAA
TCCTCACAGCTTTACGGCACAAACCTCGTTAATCTGCTGAAAC
TGTTGTGCAAAGAGAAAGACGGCAATATCACTGTTGATTTTGA
TGATGTGGTGATTCGCGGCGTGACCGTGATCCGTGCGGGCGAA
ATTACCTGGCCGGCACCGCCGATTCAGGTATCAGCTCAGCCGC
AGGCGGCACAAAAAGCGGCACCGGAAGTGAAAACTGAGGAA
AAATGTACCTGCTCACCGTGGCGTAAATACGCGTTGATGGCGC
TGGCAATCATTCTTTTTGGCTGGATGGCAAGCGTTGCGCCGAA
AGAATTCCTTGGGCACTTCACCGTTTTCGCGCTGGCCTGCGTTG
TCGGTTATTACGTGGTGTGGAATGTATCGCACGCGCTGCATAC
ACCGTTGATGTCGGTCACCAACGCGATTTCAGGGATTATTGTTG
TCGGAGCACTGTTGCAGATTGGCCAGGGCGGCTGGGTTAGCTT
CCTTAGTTTTATCGCGGTGCTTATAGCCAGCATTAATATTTTCG
GTGGCTTCACCGTGACTCAGCGCATGCTGAAAATGTTCCGCAA
AAATTAAGGGGTAACATATGTCTGGAGGATTAGTTACAGCTGC
ATACATTGTTGCCGCGATCCTGTTTATCTTCAGTCTGGCCGGTC
TTTCGAAACATGAAACGTCTCGCCAGGGTAACAACTTCGGTAT
CGCCGGGATGGCGATTGCGTTAATCGCAACCATTTTTGGACCG
GATACGGGTAATGTTGGCTGGATCTTGCTGGCGATGGTCATTG
GTGGGGCAATTGGTATCCGTCTGGCGAAGAAAGTTGAAATGA
CCGAAATGCCAGAACTGGTGGCGATCCTGCATAGCTTCGTGGG
TCTGGCGGCAGTGCTGGTTGGCTTTAACAGCTATCTGCATCATG
ACGCGGGAATGGCACCGATTCTGGTCAATATTCACCTGACGGA
AGTGTTCCTCGGTATCTTCATCGGGGCGGTAACGTTCACGGTT

	CGGTGGTGGCGTTCGGCAAACTGTGTGGCAAGATTTCGTCTAA<br>ACCATTGATGCTGCCAAACCGTCACAAAATGAACCTGGCGGCT<br>CTGGTCGTTTCCTTCCTGCTGCTGATTGTATTTGTTCGCACGGAC<br>AGCGTCGGCCTGCAAGTGCTGGCATTGCTGATAATGACCGCAA<br>TTGCGCTGGTATTCGGCTGGCATTTAGTCGCCTCCATCGGTGGT<br>GCAGATATGCCAGTGGTGGTGTCGATGCTGAACTCGTACTCCG<br>GCTGGGCGGCTGCGGCTGCGGGCTTTATGCTCAGCAACGACCT<br>GCTGATTGTGACCGGTGCGCTGGTCGGTTCTTCGGGGGCTATCC<br>TTTCTTACATTATGTGTAAGGCGATGAACCGTTCCTTTATCAGC<br>GTTATTGCGGGTGGTTTCGGCACCGACGGCTCTTCTACTGGCGA<br>TGATCAGGAAGTGGGTGAGCACCGCGAAATCACCGCAGAAGA<br>GACAGCGGAACTGCTGAAAAACTCCCATTCAGTGATCATTACT<br>CCGGGGTACGGCATGGCAGTCGCGCAGGCGCAATATCCTGTC<br>GCTGAAATTACTGAGAAATTGCGCGCTCGTGGTATTAATGTGC<br>GTTTCGGTATCCACCCGGTCGCGGGGCGTTTGCCTGGACATAT<br>GAACGTATTGCTGGCTGAAGCAAAAGTACCGTATGACATCGTG<br>CTGGAAATGGACGAGATCAATGATGACTTTGCTGATACCGATA<br>CCGTACTGGTGATTGGTGCTAACGATACGGTTAACCCGGCGGC<br>GCAGGATGATCCGAAGAGTCCGATTGCTGGTATGCCTGTGCTG<br>GAAGTGTGGAAAGCGCAGAACGTGATTGTCTTTAAACGTTCGA<br>TGAACACTGGCTATGCTGGTGTGCAAAACCCGCTGTTCTTCAA<br>GGAAAACACCCACATGCTGTTTGGTGACGCCAAAGCCAGCGT<br>GGATGCAATCCTGAAAGCTCTGTAA
GmUGT4	TTAATGTTCTCTCCACAATTCAACCAACTTATTCAATGCCATGA<br>TTGAAGAACCACCTTTTGCCATAGCTTCAGTAGCTGAAATTTTC<br>ATCTTAAAAATTCTTTGTCTGATTTCTTTACCCTTATCAGAATCC<br>ATCAATTCCATAACTCTGTCACCCAATTCTGTTGAAGAAACTA<br>AACCATCCTTATTTTGCTTAACAGCCAAACCAACCTTCATTTCT<br>TCAACCAAAATAACCTTATTCAACTTTTGTTCTGCGTACAATGG<br>CCAAGCAACCATTGGAACACCTTCACAAACTGCTTCTAAAACA<br>GAATTCCAACCACAATGAGTAACAAAACCACCAACTGAATCA<br>TGAGATAAAATTGCAGCTTGTGGAGCCCAATCTCTAACAACCA<br>AACCCTTTTCCTTTGTTCTTTCCAAGAAACCTTCTGGTAACAATT<br>CATCCAATGATGGTGGTTCACCAGAGTCACCCTCTTCGAATTCT<br>GATCTAACAACCCATAAAAATCTTTGTTCAGATTTTTCCAAACC<br>GATAGCGATTTCTCTCAATTGTGTTCTAGAAAATCTACCCATTG<br>AACCAAAAGACAAGAAAACAACTGAATGAGATGGTTGTGAAT<br>CTAACCAAGACAAACAACCATTATCATCTTTTCTACATGGAGC<br>TGAAGAAATAACTGGACCAATACAGAAAACTTTTGGTGTAGTA<br>CCTTCCATTAAACCTTCTGAAAATGCTTCAACAACTCTACCTTC<br>CATAGCTTCACAAGTATTAACAATAACACCATCAGAATCTCTC<br>ATACATGTTGCAATATCAATAAAAACTTGGTAACCTTCGTTAG<br>CTCTATCTTGCATTTGTTCTGGCAAATCATCAGTATGGATCTTTG<br>GCAAACCTGGAATAACCAAATGCATATTCAAATCTTTAAAAG

	ACTTTGTAGAGTTTTCATGGATGATGATTTGTTGCAAGAAAATT<br>GCTAAAGTTGAAGCACCAGATGTGTAGTAGAAGTAAGTTGGG<br>ATTTGCAATGCGTTAGTAACTCTTGCAGCTGAGTAGTTCATGAA<br>ATCCAAAACAATAGCTTTCAAATTAGATGTTTGTGAGATAGAG<br>TTCAAGATTCTTCTCAAATGATGACCAGTTGCTCTACACAATTC<br>AAATGTTAAAGCCATTGGTGGTAAAACAGTTGGAACAGAAAT<br>TTGTGGGATTCTATGGAATGTGATTGATGGTGTAGATGCAGTA<br>ACTGCAGCAATGTATTTTGCAGTAGCATCACATGTAAAAGCAG<br>TTGGAGATGTTGGAGTATCTTGATTTGGTGGTGGAGTCAAGAA<br>AATGATAGTGATTGACAAAGATGGATGATGTGACAAGATCAA<br>TTTACCCAATTCAACCATAGAAACCAAATGACCTCTACCCAAA<br>GCTGAGTACAAAACGATAGAATCCTTCATTGTCAT
PlUGT43	TTAAGATGTTAATTCTTGAATTAATGAAACCAAATTAGAATAT<br>GAAGAACCATTTTCTAACAAAGCACCTCTACAAGTATCTGACA<br>TTTCCTTAACCTTTCTTCTGATTTCGTCACCACCTTTCATCAAAG<br>ATCTCAAACCGTTTTCAACTTCTTCTGCTCTAACCAAATCAGCA<br>CCAACTCTGTAATCAACTGAAATTTCAACTGCTAAACCCAAAT<br>CTCTAACCATTTGGAAAGCGTTCATTTGTTGTTCAGAGTACAAT<br>GGCCATGTTGCAACTGGAACACCATGCCATAATGATTCCAAAA<br>TAGAATTCCAACCACAATGAGAAACAAAACCACCAACTGCTT<br>TATGAGCTAAAACAACAGCTTGTGGAACCCAACCACAAACCA<br>AACCGATGTTGTTAGTTCTTTCCAAGAAACCATCTGGCAAAAC<br>ATCCTTATGGTTTTCGTAATCTCTTGGGTATTCCAATTGTGCTTT<br>TGGAGATTCTCTTAAAGCCCACAAAAATCTAACACCTGCTCTT<br>TCCAAACCGATAGCGATTTCTTCAACTTGGTTAGCTTCCAATGA<br>ACCCATAGAACCGAAACACAACAAAACAACTGAAGACAATG<br>GTTGTTGATCCAACCATTCCATAATTCTTTTGTATTGTGCTGGAT<br>TTGGATCCCATTGAGCAGAACCAACCAAATCCAAAATTGGAC<br>CAATTGGATAAACTCTTTGCAATTGTGAATCGTTATGCAAAGA<br>TTGCAAAGCATGTGTTTCTAATTCTTGCAAAGTATTAATAACGA<br>TACCCTTTGTTTCCTTGTATCTTCTTGCATGGTATGACAACCAA<br>AAAGTACCATCTTTAGCATCCAAAACCAAATTTGGGAAAACA<br>GATCTTGGCAACAAATTCTTGAAACATGGAACAGTGAATTCTG<br>ACTTAGATTCTGCCAAATCGAATCTTGGCAAATCCAAAGTAAA<br>ACCCAAACATGATGCTGGAGAAGCGAAAAACAAATAACATG<br>GAACAGCCAATTCTGCAGCAACATCAATTAATGTAGTTGAGAA<br>CATATCAACGAAGATTGCAGCCAATCTAACTGAATTTGAAGAA<br>TTAGATTCTGTAGTCTTCATCAAATTCAACAATGCATGCTTAAC<br>ATGATGTTTATGGTTTTGCATATGCAAAGACAAGAAACCAACC<br>AAAGTTTGGTATTGTTCTGGAGCTGGTGGATCAACGATTGGCA<br>ATTGCAACAACTTGATGTTTGCAGCTGAAGATGCTCTAGCTTG<br>AACGTATGTGTTCATCAATGGTCTTTGTGGCATTGAAACTGTCA<br>AGATAGTTGCAGAAAATCTTGGATCATGCTTAGTCAACAAATT<br>AGCGAATTCAACTTGTGGAACCAAATTACCCAATGTTGGGATA

GCAATAAAAACAACTTCGTATCTAGTCAT

Table S5 Guide RNA sequences used in this study.

Site	Guide RNA sequences	Role
XI-5	TGTCCCTGTATACTGCAAAG	Common integration site
XI-2	GGTCCCTAAGGATATCTCGG	Common integration site
EXG1	GATCATGGAAGCTATTCCAG	Glycoside hydrolase site
OCA5	CTTCAGCAGAAATTTCACGA	Inositol pyrophosphatase
FAS1p	TTGATAAGGAAAAAGAACCA	Promoter site of the *FAS1* gene

Table S6 Plasmids used in this study.

Name	Relevant characteristics
pY26-1	*pY26-SED1p-GeHIS-Ter22-GmCPR-TDH1p-INO1p-GmHID-CYC1t*
pY26-2	*pY26-SED1p-GmIFS-Ter22-GmCPR-TDH1p-INO1p-GmHID-CYC1t*
pY26-3	*pY26-SED1p-GmIFS2-Ter22-GmCPR-TDH1p-INO1p-GmHID-CYC1t*
pY26-4	*pY26-SED1p-GuIFS-Ter22-GmCPR-TDH1p-INO1p-GmHID-CYC1t*
pY26-5	*pY26-SED1p-MtIFS-Ter22-GmCPR-TDH1p-INO1p-GmHID-CYC1t*
pY26-6	*pY26-SED1p-PlIFS-Ter22-GmCPR-TDH1p-INO1p-GmHID-CYC1t*
pY26-7	*pY26-SED1p-TpIFS-Ter22-GmCPR-TDH1p-INO1p-GmHID-CYC1t*
pY26-8	*pY26-SED1p-GeHIS-Ter22-GmCPR-TDH1p-INO1p-GeHID-CYC1t*
pY26-9	*pY26-SED1p-PlIFS-Ter22-GmCPR-TDH1p-INO1p-PlHID-CYC1t*
pY26-10	*pY26-SED1p-GmIFS-Ter22-CrCPR2-TDH1p-INO1p-GmHID-CYC1t*
pY26-11	*pY26-SED1p-GmIFS-Ter22-AtCPR-TDH1p-INO1p-GmHID-CYC1t*
pY26-12	*pY26-SED1p-GmIFS-Ter22-LjCPR-TDH1p-INO1p-GmHID-CYC1t*

pY26-13	*pY26-SED1p-GmUGT4-DIT1t*
pY26-14	*pY26-SED1p-PlUGT43-DIT1t*
pRS424-1	*pRS424-SED1p-STB5-TEr22*
pRS424-2	*pRS424-SED1p-YEF1-TEr22*
pRS424-3	*pRS424-SED1p-EcPntAB-TEr22*
pRS424-4	*pRS424-SED1p-ALD6-TEr22*
pRS424-5	*pRS424-SED1p-HEM2-TEr22-HEM3-TDH3p*
pRS424-6	*pRS424-TDH1p-PGM1-ADH1t*
pRS424-7	*pRS424-TDH1p-PGM2-ADH1t*
pRS424-8	*pRS424-TDH1p-PGM1-ADH1t-INO1p-UGP1-CYC1t*
pRS424-9	*pRS424-TDH1p-PGM2-ADH1t-INO1p-UGP1-CYC1t*
Cas-1	*Ca9 gRNA-XI5*
Cas-2	*Ca9 gRNA-XI2*
Cas-3	*Ca9 gRNA-EXG1*
Cas-4	*Ca9 gRNA-OCA5*
Cas-5	*Ca9 gRNA-FAS1*
